# Mapping of RORγt^+^ dendritic cells in human tissues establishes their preferential niche in adult lymph nodes

**DOI:** 10.3389/fimmu.2025.1527499

**Published:** 2025-05-30

**Authors:** Silvia Lonardi, Francisco MA Bianchetto-Aguilera, Matilde Monti, Anna Di Matteo, Francesco Missale, Sara Picinoli, Mattia Bugatti, Martina Benedetti, Giorgia Ferrari, Marco A. Cassatella, Daniele Moratto, Stefania Zini, Valentina Agostini, Viola Savoldi, Luisa Lorenzi, Marco Chiarini, Fabio Facchetti, Shitong Wu, Alina Ulezko Antonova, Yizhou A. Liu, Marina Cella, Claudia Ghigna, Marco Colonna, William Vermi

**Affiliations:** ^1^ Pathology Section, Department of Molecular and Translational Medicine, University of Brescia, Brescia, Italy; ^2^ General Pathology Section, Department of Medicine, University of Verona, Verona, Italy; ^3^ Istituto di Genetica Molecolare “Luigi Luca Cavalli-Sforza”, Consiglio Nazionale delle Ricerche, Pavia, Italy; ^4^ Department of Otorhinolaryngology, Head and Neck Surgery, Maastricht University Medical Center, Maastricht, Netherlands; ^5^ GROW—Research Institute for Oncology and Reproduction, Maastricht University, Maastricht, Netherlands; ^6^ Clinical Chemistry Laboratory/Diagnostic Department, Azienda Socio Sanitaria Territoriale (ASST) Spedali Civili di Brescia, Brescia, Italy; ^7^ Department of Pathology and Immunology, Washington University School of Medicine, St. Louis, MO, United States

**Keywords:** RORγt, RORC2, dendritic cells, lymph nodes, lymphoid organs

## Abstract

**Background:**

The retinoic-acid-receptor-related orphan receptor gamma t (RORγt) isoform is required for the development of lymphoid organs, T-helper 17 cells (Th17), and innate lymphoid cells (ILC3s). Recent data in mouse and human have revealed non-T, non-ILC3 cell populations with antigen presenting features that express RORγt. This study maps the presence of RORγt cells with dendritic cell (DC) features in human adult and fetal lymphoid tissues.

**Methods:**

By combining multicolor flow cytometry, RNAseq of peripheral blood cells, analysis of lymph node scRNAseq datasets, and microscopic analysis on human tissue sections and single-cell suspensions, this study maps the presence of RORγt cells with DC features in human tissues.

**Results:**

RORγt-DCs are found in human lymphoid organs, particularly in lymph nodes. Lymph node RORγt-DCs are located in the interfollicular area surrounding high endothelial venules and in the marginal sinuses. In terms of phenotype, RORγt-DCs are distinct from other nodal dendritic cells. They express PRDM16 and PIGR as well as transcripts supporting the antigen presentation machinery, while lacking stromal markers. A significant fraction of RORγt-DCs is proliferating, suggesting local self-renewal. Moreover, most of them lack autoimmune regulator (AIRE) expression. Comparison with mouse RORγt Thetis cells (TC) and Janus cells (JC) shows more similarity with group II TC than group I and III TC or JC, all of which are tolerogenic and express AIRE.

**Conclusion:**

Overall, this study identifies human lymph nodes as a relevant niche for RORγt-DCs and establishes tools for their microscopic mapping in human disease states.

## Introduction

The retinoic-acid-related orphan receptor RORγt is an isoform of the nuclear hormone receptor RORγ that was originally identified in thymocytes [reviewed in (1, 2)]. RORγt has an essential role in T-cell development, promoting the survival of double positive thymocytes through induction of B-cell lymphoma-extra large (BCL-XL) (3, 4). RORγt is also necessary for the development of lymphoid tissue inducer (LTi) cells (5), which initiate the formation of secondary lymphoid organs (SLO) (5, 6) and Peyer’s patches (PP) (6–8). Within the lymphoid compartment, RORγt is also a lineage-specifying transcription factor for Th17/Tc17 (9–11) and for group 3 innate lymphoid cells (ILC3s) (12–15). ILC3s include LTi-like cells that support tertiary lymphoid organs and produce IL-17, as well as natural cytotoxicity receptor (NCR)^+^ ILC3s, which produce interleukin (IL)-22, granulocyte–macrophage colony-stimulating factor (GM-CSF), and tumor necrosis factor (TNF), sustaining innate and adaptive responses especially at mucosal barriers (16, 17).

Recent findings have unexpectedly shown that RORγt is also expressed in subsets of antigen-presenting cells with dendritic cell (DC) features. DCs shape adaptive immune responses through detection, capture, and presentation of antigens (18). Both tissue-resident and circulating DCs are highly heterogeneous and consist of multiple specialized subtypes, underscoring their pivotal role in orchestrating both local and systemic immune responses. This diversity can be categorized based on lineage, localization, transcriptional programs, and functional states (19). Recent -omics and functional studies have further expanded on the level of DC heterogeneity identifying six specialized subtypes (20). The multifaceted functions of DCs are also acquired through a maturation process, during which they evolve into specialized states. Upon activation of pattern recognition receptors, DCs undergo maturation, which includes the induction of the antigen-processing machinery, a balanced expression of co-stimulatory and co-inhibitory molecules, metabolic reprogramming, and the production of immunostimulatory cytokines. Mature DCs then migrate to micro-anatomical niches, including secondary lymphoid organs and tertiary lymphoid structures (TLS), where they efficiently prime T cells and fine-tune their differentiation and activation.

RORγt positive cells with antigen-presenting features have been recently identified. These include Thetis cells (TC) and Janus cells (JC) (21–24) in the mouse, and RORγt-DC in human tissues (25). TC display a DC–medullary thymic epithelial cells (mTEC) hybrid phenotype, showing also a significant transcriptional overlap with ILC3 and include four different subsets (24, 26, 27). Two of these, TCI and TCIII, express the autoimmune regulator AIRE. JC have an mTEC phenotype, express AIRE, and may in fact correspond to TCI/III. Both TC and JC have been implicated in the development of RORγt^+^ peripheral regulatory T cells (pTreg) that maintain tolerance to microflora antigens. Human RORγt-DC co-express a set of DC and ILC3 markers and are closely related to a subset of conventional DCs called DC2. RORγt-DCs have been found in the intestinal and tonsillar mucosae and have been shown to stimulate the proliferation of T cells that express CCR8, a marker of human Treg. Here, we systematically examined the distribution of RORγt-DC in healthy and diseased human tissues by immunohistochemistry (IHC) and immunofluorescence microscopy using specific markers. We complement these studies with bioinformatic analysis of available scRNAseq databases. Results demonstrate that RORγt-DCs are present in specific locations of primary and secondary lymphoid organs and exhibit dendritic cell features.

## Materials and methods

### Human tissues

Formalin fixed paraffin embedded (FFPE) tissue blocks used for this study were retrieved from the tissue bank of the Department of Pathology (ASST Spedali Civili di Brescia, Brescia, Italy). Human tissues included reactive lymph nodes (n=104; **Supplementary Tables S1**, **S2**), bone marrow, spleen, thymus and tonsils (n= 60; **Supplementary Table S3**), non-lymphoid normal tissues (n=53; **Supplementary Table S4**), and fetal lymph nodes (n=41; **Supplementary Table S5**) from seven autopsies. For cell suspension used for flow cytometry (FC) analysis, a 1–10-mm^3^ fragment of the lymph node biopsy (n=5) was finely minced with a scalpel on a 100-mm cell culture dish. Tissue dissociation into single-cell suspension was accomplished using gentleMACS™ Dissociator (Miltenyi Biotec). The suspension was filtered onto a 40-µm cell strainer and centrifuged at 1200 RPM for 5 min. Red blood cell lysis was performed using 1× RBC Lysis Buffer (BioLegend, #420301) following the manufacturer’s instructions. Finally, cell suspensions were suspended in fetal bovine serum (FBS) with 10% dimethyl sulfoxide (DMSO) and stored in liquid nitrogen.

### Immunohistochemistry

Four-micron-thick tissue sections were used for immunohistochemical staining. RORγt (clone 6F3,1, diluted 1:1500 from Merck) was revealed after 40 min in ethylenediaminetetraacetic acid (EDTA) buffer pH 8.0 as antigen retrieval using Novolink Polymer (Leica Microsystems, #RE7280-CE) followed by 3,3’-diaminobenzidine (DAB) as chromogen. For double immunohistochemistry, RORγt antibody was coupled with a panel of markers detailed in **Supplementary Table S6**. Briefly after completing the first immune reaction, the second was visualized using Mach 4 MR-AP (Biocare Medical, #M4U536), followed by Ferangi Blue (Biocare Medical, #FB813) as chromogen. For triple immunohistochemistry, the third antigen was revealed using Mach 4 MR-AP and StayRED/AP (Abcam, #ab103741) as chromogen. For quadruple immunohistochemistry, the third antigen was revealed using Novolink Polymer followed by 3-amino-9-ethylcarbazole (AEC, Vector Laboratories #30013) and the fourth antigen using Mach 4 MR-AP followed by StayRED/AP (Abcam, #ab103741) as chromogen.

Isotype control (Mouse IgG2a) staining was performed on reactive lymph node. Sequential stainings were also performed to recognize the transcription factors and RORγt as previously described (28).

### Multiplex immunofluorescence staining

Immunofluorescence was performed with a tyramide signal amplification system on 2 μm FFPE. The antigen retrieval was performed using microwave in EDTA pH 8.00 or TRIS-EDTA pH 9.00 buffers. Aspecific binding was prevented through incubation with BSA (5% in TBS/Tween). Primary antibodies were incubated 1 h RT (**Supplementary Table S6**) and revealed using Novolink Polymer (Leica Biosystems, #RE7280-CE) followed by tyramide fluorophore AF488 (Thermo Fisher Scientific, #B40953), CF555 (Biotium #96021), and CF647 (Biotium #96022). After completing the first immune reaction, the second and the third were visualized combining the different tyramide fluorophores.

Sections were digitized using Axioscan7 (Zeiss) at 20× magnification using Colibri 7 lamp as LED light source with different illumination wavelength (450–488; 540–570; 615–648) depending on the different modules of the LED (385 nm, 475 nm, 567 nm, and 630 nm).

### Digital image analysis

RORγt^+^ nuclei diameters were obtained using the ruler tool of ImageScope (Leica Microsystems). The nuclear areas of RORγt^+^CD3^−^ (n=100 cells) and RORγt^+^CD3^+^ (n=100 cells) were measured on digitalized slides from reactive lymph nodes (n=10) double stained for RORγt and CD3, using the Positive Pixel count algorithm (ImageScope). Proliferation rate of PIGR^+^ cells was defined as a fraction of PIGR^+^Ki-67^+^ cells on total PIGR^+^ cells in reactive lymph nodes (n=5). Consecutive stains on the same section were performed to characterize the morphology of RORγt^+^CD3− cells; briefly, the section was stained for H&E, scanned, and stained for RORγt; reacquired, virtual slides were then realigned.

Digitalized immunofluorescence slides were analyzed using QuPath-0.4.3; briefly, at first RORγt signals were used as a nuclear marker to detect the cells, and then a single measurement classifier was trained to detect CD3, CD127, and RORγt positivity. At last, a composite classifier was used to identify the different cell populations; because CD127 stain overlaps with CD3, the RORγt^+^CD127^+^ population was defined by only CD3^−^ cells. The RORγt^+^ cell populations were graphed for density/mm^2^ (the lymph node areas were obtained by digital image selection), percentage of all nucleated cells (H&E digitalized slides were processed using IHC nuclear algorithm to count all nodal cells as shown in **Supplementary Figure S2E**), or percentage of the single population of total counted RORγt^+^ cells as well as absolute number in the lymph node.

Absolute count/mm^2^ of a cohort of cases (n=25, five areas each randomly chosen) was performed preliminarily to establish quantification categories (+= 0.01–5 cells/mm^2^, ++= 6–25 cells/mm^2^, +++= >26 cells/mm^2^). The perimeter was drawn on digitalized slides through ImageScope to achieve the total nodal area in mm^2^.

RORγt-DC lymph nodes (n=4, score +++) triple and quadruple stained on serial sections for CLA/Heca 452 and dendritic cell markers were digitalized and synchronized. Drawn squares of determined area of 0.025 mm^2^ were cut and pasted around high endothelial venules (HEV; n=15) with RORγt-DC enrichment to count the major dendritic cell populations nearby.

### Western blot

Cell lysates from HEK293T were obtained from cells seeded in a 24-well plate in complete DMEM medium. Cell lysates from thymocytes and lymph node cell suspensions were obtained from 2×10^6^ cells. Cells were lysed in RIPA lysis buffer (Thermo Fisher Scientific, #89900) supplemented with Protease Inhibitor Cocktail (Merck, #78440) and incubated in ice for 20 min. Cell lysates were centrifuged at 13,000 RPM for 15 min, and the suspension was collected and stored at −20°C. Protein concentration was determined by Bradford assay, and 15 µg of total proteins was loaded on 4%–12% NuPAGE^®^ Bis–Tris Mini Gels (Invitrogen, #NP0335) under reducing condition and transferred onto a PVDF membrane (Invitrogen, #LC2007). Membranes were blocked with 5% milk (Biotium, #22012) in TBS-T (TBS with 0.05% Tween 20; Invitrogen, #28360) for 1 h at room temperature. Primary antibodies to RORγt (clone 6F3.1, diluted 1:1000 from Merck) and Histone H3 (polyclonal, diluted 1:5000, Merck, #06-755) were incubated o/n at 4°C in TBS-T with 5% BSA (Merck, #A3059). The anti-rabbit (Thermo Fisher Scientific, #31460) or the anti-mouse (Cell Signaling Technology, #7076) secondary antibodies conjugated with horseradish peroxidase were incubated for 1 h at room temperature. Detection was performed using the SuperSignal™ West Pico Chemiluminescent Substrate (Thermo Fisher Scientific, #34577) and visualized by autoradiography.

### Flow cytometry analysis

Analysis of RORγt expression was performed on immune cells starting from three healthy donors’ peripheral blood (PB) samples and from five frozen lymph nodes (LN) cell suspensions. Specifically, 250 µL of whole blood were lysed with 2 mL of FACS Lysing Solution (BD Biosciences, #349202), and at least 5×10^5^ cells/tube of LN suspension were stained with Far Red Live/Dead Fixable Stain kit (Thermo Fisher Scientific, #L10120) following the manufacturer’s instructions. Then, cells were incubated with appropriate mixes of fluorochrome-conjugated antibodies to identify target cell subsets (**Supplementary Table S6**). For all panels, intracellular staining was performed to identify RORγt-expressing cells using the Transcription Factor (TF) Buffer Set (BD Biosciences, #562574), according to the manufacturer’s instructions. Specifically, the unconjugated mouse IgG2a anti-human ROR gammaT monoclonal antibody (Merck, #MABF81) was used as the primary antibody, followed by a PE-conjugated goat anti-mouse IgG2a secondary antibody (Southern Biotech, #1080-09). A minimum of 2×10^5^ CD45^+^ gated leukocytes were acquired and identified based on the forward light scatter (FSC) versus side light scatter (SSC) profile and singlets selection. Lymphocytes, monocytes, granulocytes, innate lymphoid cells (ILCs), and dendritic cell subsets were identified using gating strategies, as follows, while the fluorescence minus one (FMO) controls were used for all the immune cell subsets tested to discriminate between background fluorescence and RORγt2 positive cells.

Lymphocytes: CD4^+^ or CD8^+^ T-cell subsets were gated on CD45^+^CD3^+^ cells. Th17 cells were identified as CCR6^+^CD161^+^ double positive cells gated on CD4^+^CD45RA^−^ cells. Similarly, CD45RA^−^CCR6^+^CD161^+^ cells were selected within CD8^+^ T cells. Analysis of RORγt expression was performed also on CD45^+^CD3^−^ lymphocytes, which comprised CD19^+^ B cells, CD56^+^ CD16^+/−^NK cells. Monocytes: CD14/CD16 cells were gated as CD4^+^HLA-DR^+^ cells on the lineage negative (Lin^−^; CD19^−^CD3^−^CD15^−^) fraction and comprised classic CD14^+^CD16^−^, non-classic CD16^+^CD14^−^, and intermediate CD16^+^CD14^+^ monocytes. Granulocytes: CD16^+^ population was gated on SSC^++^CD15^+^ PMN cells. Dendritic cells: DC subsets were defined as CD4^+^HLA-DR^+^ mononuclear cells on Lin^−^ (CD3^−^CD14^−^CD16^−^CD19^−^) gate. Three different DC subsets were then gated according to expression of cell surface specific markers: CD141^+^ for cDC1, CD1C^+^ for cDC2, and CD303^+^ for pDC cells, respectively. Samples were processed on a FACSCanto II flow cytometer (BD Biosciences). Datasets were analyzed using the FlowJo X software (Tree Star Inc.) and FACSDIVA software (BD Biosciences).

### RORγ1/RORC1 and RORγt/RORC2 mRNA expression

Isolation of total RNA was carried out on cell suspensions from lymph nodes and tonsils with an RNeasy mini kit (Qiagen, #74104) according to the manufacturer’s protocol. The cDNA was synthesized from 500 ng total RNA using the qScriberTM cDNA Synthesis Kit 5× and 20× (TwinHelix, #RTK0104). *RORγ1/RORC1* and *RORγt/RORC2* expression analyses were performed by RT-qPCR technology using the Gene Expression Assay RORC RefSeqNM_001001523/NM_005060 (Hs01076112_m1, Thermo Fisher Scientific, #4453320) and RORC RefseqNM_001001523 (Hs02892670_m1, Thermo Fisher Scientific, #4448892) in combination with TaqMan^®^ Universal Master Mix II (Thermo Fisher Scientific, #4364340). The glyceraldehyde-3-phosphate dehydrogenase (GAPDH; Thermo Fisher Scientific, #4331182Hs02758991-g1) transcript was used as normalization control. The results were obtained using the Vii-A-7 Real-Time PCR System and analyzed using QuantStudio™ Real-Time PCR software (Thermo Fisher Scientific). The threshold cycle (Ct) was determined for each sample, and quantification was performed using the comparative Ct method. ΔCt was derived as Ct Target − Ct Housekeeping.

### RNAscope *in situ* hybridization

To localize *RORγt/RORC2* and *PRMD16* transcripts by RNAscope assay (Advanced Cell Diagnostics), we used RNAscope 2.5 HD Assay-RED, Hs-RORGT probe (#311061) recognizing the nt 152 to 744 of the RORγt reference sequence NM_001001523 and Hs-PRMD16 (#581961) recognizing the nt 1633–3244 of the *PRMD16* reference sequence NM_022114.4. Sections from fixed human tissue blocks were treated following the manufacturer’s instructions, as previously reported (25). Briefly, freshly cut 3-μm sections were deparaffinized in xylene and treated with the peroxidase block solution for 10 min at room temperature followed by the retrieval solution for 15 min at 98°C and by protease plus at 40°C for 30 min. The hybridization was performed for 2 h at 40°C. The signal was revealed using RNAscope 2.5 HD Detection Reagent and FAST RED. Control probes included Hs-POLR2a-C2 (#310451) and dapB-C2 (#310043-C2). For RNAscope and immunohistochemistry, dual stain detections by RNAscope were followed by immunoreaction visualized using Novolink Polymer (Leica Microsystems, #RE7280-CE) followed by DAB or using Mach 4 MR-AP (Biocare Medical, #M4U536) followed by Ferangi Blue (Biocare Medical, #FB813). For RNAscope and immunohistochemistry triple stain, we used the Co-detection Ancillary kit (Advanced Cell Diagnostics, #323180) following the manufacturer’s instructions. Briefly, the antibody was diluted using the Co-detection antibody diluent and incubated overnight. RNAscope proceeded as usual after a step of fixation (using buffered formalin), and after its revelation, a Co-detection blocker was used followed by Novolink polymer and DAB. The third stain was obtained by incubating the last antibody overnight and revealing it through Mach 4 MR-AP followed by Ferangi Blue.

### Bioinformatic analysis of RORγ1/RORC1 and RORγt/RORC2 expression

To quantify the usage of each isoform, we used the intermediate output files from *vast-tools* (eej2 files) for all sample available in VastDB for hg38 (29, 30). These files contain the number of read counts mapping to each exon–exon junction. Thus, to obtain the percent of each isoform, we used the following exon–exon junction read counts: RORγ1 (exons 2γ-3) and RORγt isoform (exons 1γt-3). Only samples with a minimum of 10 read counts in total were considered.

### RORγ1/RORC1 and RORγt/RORC2 transcript quantification from human PBMC datasets

The RNAseq datasets used in this study were downloaded from the Gene Expression Omnibus database (http://www.ncbi.nlm.nih.gov/geo/) under the following accession numbers:

GSE107011 for Th17 and MAIT cells (31) and GSE136107 for tonsil slan^+^ cells, type 2 conventional CD1C^+^ DCs (cDC2) and CD11B^+^CD14^+^ macrophages (MΦ), as well as peripheral cDC2, classical (CL), intermediate (INT), NC, and slan^+^ NC monocytes (32). In brief, contaminant adapters in the fastq files were detected using FastQC v0.12.1. Subsequently, the removal of adapters and base quality trimming were performed using Trim Galore (v 0.6.19) with the length parameter set to 50. The quantification of transcript isoforms was performed by Salmon (v1.2.0) using a decoy-aware transcriptome (human transcriptome Ensembl version 110) in pseudo-alignment mode and the following settings: library type (−l A), correction of sequence-specific biases (−-seqBias) and position-specific biases (−-posBias). Additionally, Salmon provided the values of transcripts per million (TPM) which were then imported into R using the tximport package with the “dtuScaledTPM” scaling method and visualized using the ggplot2 R package.

### Bioinformatic analysis of scRNAseq data of human lymph nodes

Data analyses were performed using the Seurat R package (v4.0.4). Cells with less than 1000 and more than 5000 unique detected features were excluded. Prior to analysis, unwanted cell types were removed from the dataset using cell type specific markers. Normalization was achieved using log transformation. Following normalization, highly variable genes were identified and selected for Principal Component Analysis (PCA) reduction of high-dimensional data and for graph-based clustering analysis. The resolution in the FindClusters function was set at values between 0.2 and 0.5, and the clustering results were shown in a computed Uniform Manifold Approximation and Projection (UMAP) plot (dims = 1:7). Different cell types were grouped and annotated based on cell type defining markers. Selected signature genes were constructed as follows: cDC1: XCR1, BTLA, BATF3, CLEC9A; cDC2: CD1C, NOTCH2, SIRPA, CD14; RORγt-DC: RORC, PIGR, PRDM16, CLEC4A. Differential expression testing was done using non-parametric Wilcoxon rank sum test. Dot plots were generated using the DotPlot function in Seurat. Violin plots were generated using the VlnPlot function in Seurat. Volcano plots were generated using the EnhancedVolcano function in Seurat.

### Statistical analysis

Standard descriptive statistic was used for data summarizing, expressing means, medians, interquartile range, and ranges. Kruskal–Wallis test was used for group comparisons in quantitative variables, and Fisher’s exact test for qualitative ones, as appropriate. A heatmap was built for graphical evaluation of the results. In all the analyses, a significance level of 5% was used. R (version 4.4.1) and R studio were used for statistical analysis.

### Study approval

Human samples were obtained following informed written consent. All experimental protocols were approved by the local IRB (WV, institutional review board code NP906; FF, institutional review board code NP 3719). The methods were carried out in accordance with the approved guidelines. All analyses of human samples, including retrospective analysis of archival material, were conducted in compliance with the Declaration of Helsinki and with policies approved by the local IRB.

## Results

### RORγt expression is restricted to human lymphoid organs

**Figure 1:**
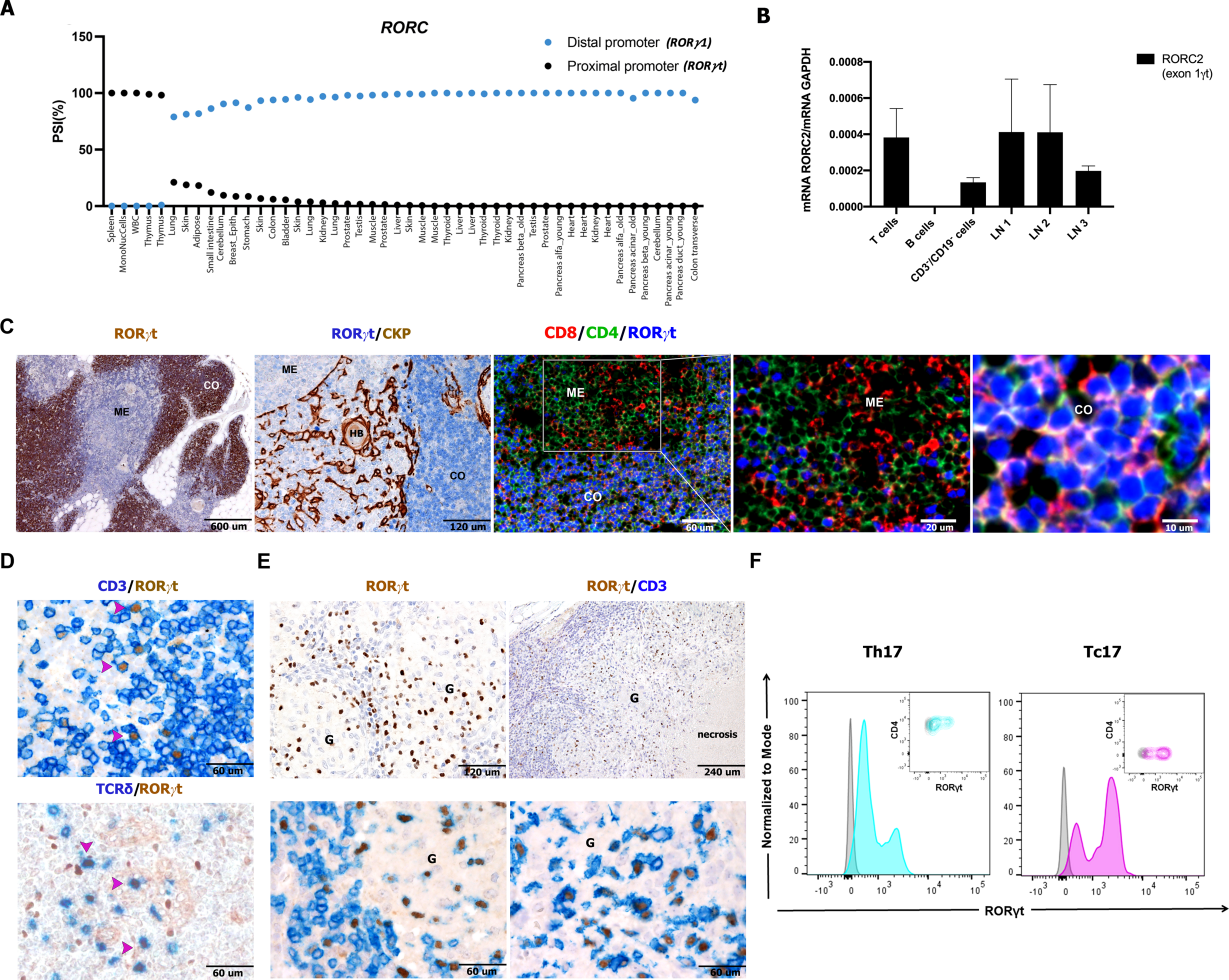
Detection of RORC2 transcript and RORγt protein in human lymphoid tissues and lymph nodes cell suspensions. **(A)** VastDB analysis showing the percentage “spliced in” for RORγ1 (blue points) and RORγt (black points) isoforms in the indicated tissues. **(B)** RT-qPCR analysis by using a TaqMan probe specific for the RORγt variant in T cells, B cells, and non-T/non-B fraction (CD3^−^/CD19^−^ cells) sorted from tonsil and in three lymph node suspensions (LN1, 2, 3). The RORγt expression was normalized to GAPDH expression (−ΔCt) using the comparative Ct method and 2^−ΔCt^ values are shown. Bars represent the mean ± SD (n = 2). Sections are from formalin fixed paraffin embedded (FFPE) human thymus **(C)**, lymph nodes (**D**, n=5), and granulomatous lymphadenitis **(E)** and stained as indicated by labels. RORγt expression is observed (using anti-RORγt antibody, clone 6F3.1) in the cortical area (CO) on CD4^+^CD8^+^ cortical thymocytes and more occasionally in the medulla (ME). **(D)** RORγt^+^CD3^+^ T cells (also γδ T cells) are detected in FFPE tissues from reactive lymph nodes as well as from granulomatous non-necrotizing (from sarcoidosis on the left, n=3) and necrotizing lymphadenitis (from mycobacterial infection on the right, n=7) **(E)**. **(F)** Representative histogram and contour plot panels illustrate RORγt expression, as seen as fluorescence intensity (x-axis), in Th17 cells (turquoise) selected as CD3^+^CD4^+^CD45RA^−^CCR6^+^CD161^+^, and Tc17 cells (violet) selected as CD3^+^CD4^−^CD45RA^−^CCR6^+^CD161^+^, evaluated from a cell suspension of a non-necrotizing granulomatous lymphadenitis. Gray histograms and contour plots represent FMO control signal for each population tested. HB, Hassall bodies; HEV, high endothelial venules; G, granuloma.

Mouse studies have shown that the RORγt isoform encoded by the *Rorc* locus is restricted to distinct immune cell types, while the RORγ isoform is expressed in many tissues, such as liver, adipose tissue, skeletal muscle, and kidney (33) (**Supplementary Figure S1A**). The tissue distribution of human variants encoded by the *RORC* locus is not as defined. We noted that the human *RORC* gene encodes a set of transcription variants, with three of them encoding proteins (from ensemble genome browser https://www.ensembl.org/index.html) (34). The full-length *RORγ1/RORC1* (variant 1, isoform 1 or a) generates a 518 aa protein; the *RORγt/RORC2* (variant 2, isoform b or 2) encodes a shorter isoform of 497 aa with a different N-terminus region; a third transcript is generated by skipping of exon 3 and encodes for a putative protein of 423 aa, the function of which has never been investigated (**Supplementary Figure S1B**). To examine the tissue distribution of these isoforms, we studied the VastDB RNAseq data (https://vastdb.crg.eu/wiki/Main_Page). We found that the proximal promoter giving rise to the *RORγt/RORC2* variant (blue in **Supplementary Figure S1B**) is predominantly active in lymphoid tissues (**Figure 1A**), while the distal promoter giving rise to RORγ1/RORC1 is preferred in most non-lymphoid tissues (**Supplementary Figure S1B**; **Figure 1A**). This is in line with the observation that RORγt/RORC2 is restricted to immune cells (34). To validate and extend these findings to human lymphoid organs, we tested predesigned TaqMan probes specific for the *RORγt/RORC2* transcript. This analysis confirmed the expression of *RORγt/RORC2* transcript in RNA extracted from reactive lymph nodes and tonsils. Moreover, analysis of RNA from fractions of cell suspensions of human tonsils revealed RORγt expression in sorted T cells as well as in the lymphoid-depleted fraction (likely containing ILC3 and non-ILC3 innate cells), whereas the B-cell fraction rendered negative results (**Figure 1B**).

In a recent study, we mapped the distribution of RORγt^+^ cells in small intestine and tonsils using the antibody clone 6F3.1 suitable for formalin-fixed tissues (25). Here we extended this analysis to various human tissues. Clone 6F3.1 displayed a strong reactivity in the large majority of human cortical thymocytes, whereas thymus epithelial and stromal cells as well as most medullary lymphoid cells rendered negative results (**Figure 1C**). In the human thymus, RORγt expression has been recently shown in double positive (DP) thymocytes using single-cell genomic approaches (35). Accordingly, in multiplex immunofluorescence on fixed human thymus (n=3), the anti-RORγt 6F3.1 confirmed the nuclear RORγt protein positivity in DP thymocytes (**Figure 1C**). Western blot analysis with the same antibody confirmed high abundance of the RORγt protein in a primary thymocyte suspension, whereas lower amounts were detected in reactive lymph nodes (**Supplementary Figure S1C**).

We further studied RORγt expression at the single-cell level in human lymph node suspensions and sections (**Figures 1D–F**). RORγt was regularly present in lymph nodes in a fraction of CD3^+^ T cells including a subset of γδ T cells, whereas the remaining lymphoid and stromal cells rendered negative results (**Figure 1D**). Lymph node RORγt^+^CD3^+^ T cells were particularly abundant within granulomas in sarcoidosis and mycobacterial infection (**Figure 1E**), two conditions recognized as IL-17-related disorders because of the infiltration by Th17 and Tc17 (36). Flow cytometry (FC) of single-cell suspension of toxoplasma lymphadenitis by clone 6F3.1 also identified numerous CD45^+^RORγt^+^CD3^+^ CD4^+^ T cells and CD45^+^RORγt^+^CD3^+^CD4^–^ T cells (**Figure 1F**), which corresponded to Th17 and Tc17 based on their expression of CCR6 and CD161. Altogether, these findings indicate that nodal T-cell populations expressing the RORγt are specifically traced in human archival tissues.

### Nodal RORγt^+^CD3^−^ cells include ILC3 and HLA-DR^+^ non-lymphoid cells

**Figure 2:**
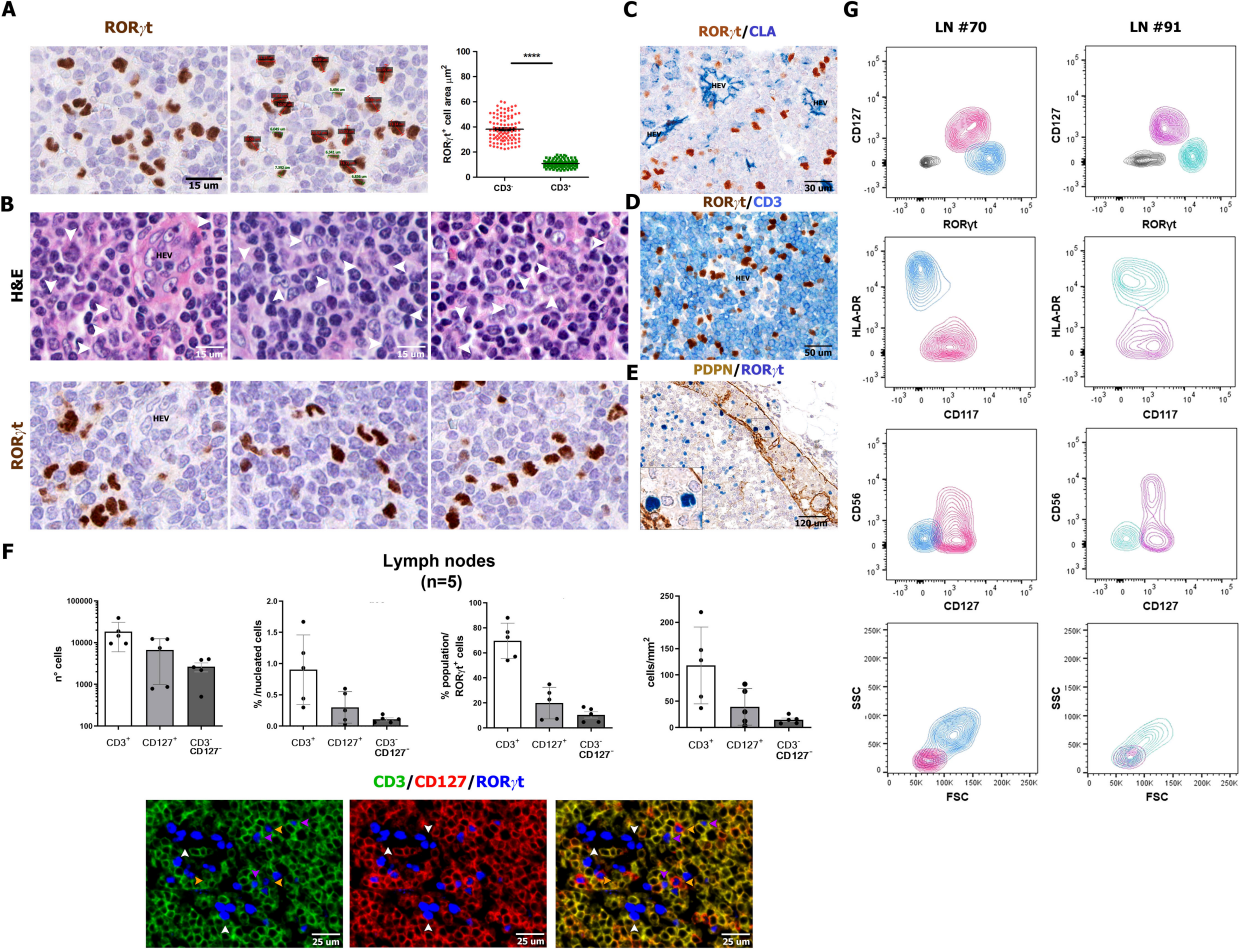
Size, morphology, and frequency of RORγt^+^CD3^−^CD127^−^ cells. Sections are from human reactive lymph nodes (n=5) **(A-F)** and stained as labeled. RORγt identifies small and round lymphoid-like cells [green in **(A)**], admixed to larger irregular cells [red in **(A)**], as confirmed by nuclear size reported in the graph p<0.0001=****. **(B)** Sequential stains for H&E and RORγt performed on the same section highlight the heterogenous morphology and irregular nuclear shape of RORγt^+^CD3^−^ cells (white arrows in the upper panels highlight the correspondent RORγt^+^ cells in the bottom panels). **(C, D)** RORγt^+^CD3^−^ cells are localized in the interfollicular area as highlighted by CLA^+^ HEVs; large RORγt^+^ cells are also found within the marginal sinus as highlighted by PDPN stain of sinus endothelial cells **(E)**. Histograms **(F)** show absolute and relative count of RORγt^+^CD3^+^ T cells, RORγt^+^CD127^+^ ILC3 cells and RORγt^+^CD3^−^CD127^−^ cells quantified in tissue sections of lymph nodes (n=5) stained and digitalized through QuPath algorithm. Graphs shows the following in order: absolute number of counted RORγt^+^ cells (error bars indicated; logarithmic scale is used), percentage on total nucleated cells (see Methods for details), relative percentage of the three RORγt^+^ populations on total RORγt^+^ cells and density/mm^2^. [**(F)** bottom panel] IF staining illustrates RORγt^+^CD3^−^CD127^−^ showing DC-like morphology (white arrow); RORγt^+^CD3^+^ T cells (fuchsia arrows) and RORγt^+^CD127^+^ ILC3 cells (yellow arrows). **(G)** RORγt^+^Lin^−^ populations on two representative human reactive lymph nodes (left and right panels) cell suspensions by flow cytometry (details in **Supplementary Table S1**). Contour plots illustrate the presence, within Lin^−^CD45^dim/+^gated cells, of RORγt^+^CD127^+^ ILC3 cells (fuchsia/pink) and RORγt^+^CD127^−^ DC-like cells (light blue/turquoise). RORγt^+^CD127^+^ ILC3 cells are CD117^+^HLA-DR^−^, also including a CD56^+^ subset, whereas RORγt^+^CD127^−^ DC-like cells are HLA-DR^+^CD117^−/dim^. According to the FSC/SSC plot, RORγt^+^ ILC3 are of lymphoid morphology (FSC^low^SSC^low^), whereas the majority of RORγt^+^ DC-like cells display monocyte-like morphology (FSC^mid^SSC^mid^).

In human lymph nodes, a bi-modal spectrum of distribution of the RORγt^+^ cells was found, both in terms of signal intensity and nuclear morphometry (size and morphology). Specifically, in addition to positive small round cells showing weak expression of RORγt and corresponding to T cells, we could also detect a stronger nuclear reactivity for RORγt in cells showing a more heterogeneous morphology also including DC-like cells with large nuclei, with variable shape ranging from kidney-like to polylobate, folded or indented (**Figures 2A, B**). These cells were localized in the inter-follicular area (**Supplementary Figure S2A**). They were found nearby the high endothelial venules (**Figures 2C, D**; **Supplementary Figure S2A**), as confirmed by double stain for RORγt and CLA/Heca 452 (**Figure 2C**), and in the marginal sinuses, as shown by co-stain with PDPN (**Figure 2E**). These cells were detectable in a large set of lymph nodes (n=104), throughout all ages in reactive or pathological patterns (**Supplementary Figure S2B** and **Supplementary Tables S1**, **S2**); among relevant association with the variables of the cohort, we found that this population was particularly enriched in larger lymph nodes showing reactive follicular hyperplasia, whereas their frequency was reduced in granulomatous lymphadenitis (**Supplementary Figure S2B**, **Supplementary Tables S1**, **S2**).

By multiplex immunostaining, a fraction of RORγt^+^CD3^−^ cells were positive for CD45^+^ (**Supplementary Figure S2C**): these cells encompassed a variable combination of ILC3 and non-ILC3 cells, based on the expression of CD127, CD117, and CD56 (**Supplementary Figure S2D**). Digital image quantification on triple immunofluorescence staining (n=5; **Figure 2F**; **Supplementary Figure S2E**) showed that RORγt^+^CD3^−^CD127^−^ non-ILC3 cells represented a very small fraction of nodal cells (**Supplementary Figure S2F**) and a minor percentage of RORγt^+^ cells (**Figure 2F**). To further support this observation, we tested anti-RORγt antibody on single-cell suspensions from reactive lymph nodes (n=4) by multiparametric flow cytometry. Within CD45^+^ cells, flow cytometry identified lin^−^CD127^+^CD117^+^HLA-DR^−^RORγt^+^ ILC3s (mean ± SD: 34.32% ± 21.16%), including a CD56^+^ subset consistent with NCR^+^ ILC3. RORγt^+^ ILC3s displayed an evident lymphoid-like morphology according to forward (FSC) and side scatter (SSC) (**Figure 2G**). A second population of lin^−^CD127^−^CD117^−^HLA-DR^+^RORγt^+^ cells were also recognized (mean ± SD: 62.30% ± 21.74%), showing monocytoid features, according to their size and granularity by FSC and SSC (**Figure 2G**). By expanding their phenotypic characterization (n=4) on tissue sections, we could confirm that most nodal RORγt^+^CD3^−^ cells lack lineage markers γδ and αβ TCR chains, CD20, CD56, CD16, and CD14 (**Supplementarys Figure S3A-F**), as well as of markers of follicular dendritic cells (CXCL13 and PDPN; **Supplementary Figures S3G, H**), fibroblastic reticulum cells (SMA and CXCL12; **Supplementary Figures S3I, J**), and endothelial cells (CD31 and CD34; **Supplementary Figures S3K, L**). In conclusion, flow cytometry and immunohistochemistry identify a small fraction of HLA-DR^+^RORγt^+^ non-ILC3 cells in the lymph nodes of a large cohort of cases, which likely correspond to RORγt-DCs.

### Nodal RORγt^+^HLADR^+^ cells correspond to dendritic cells expressing PIGR and PRDM16

**Figure 3:**
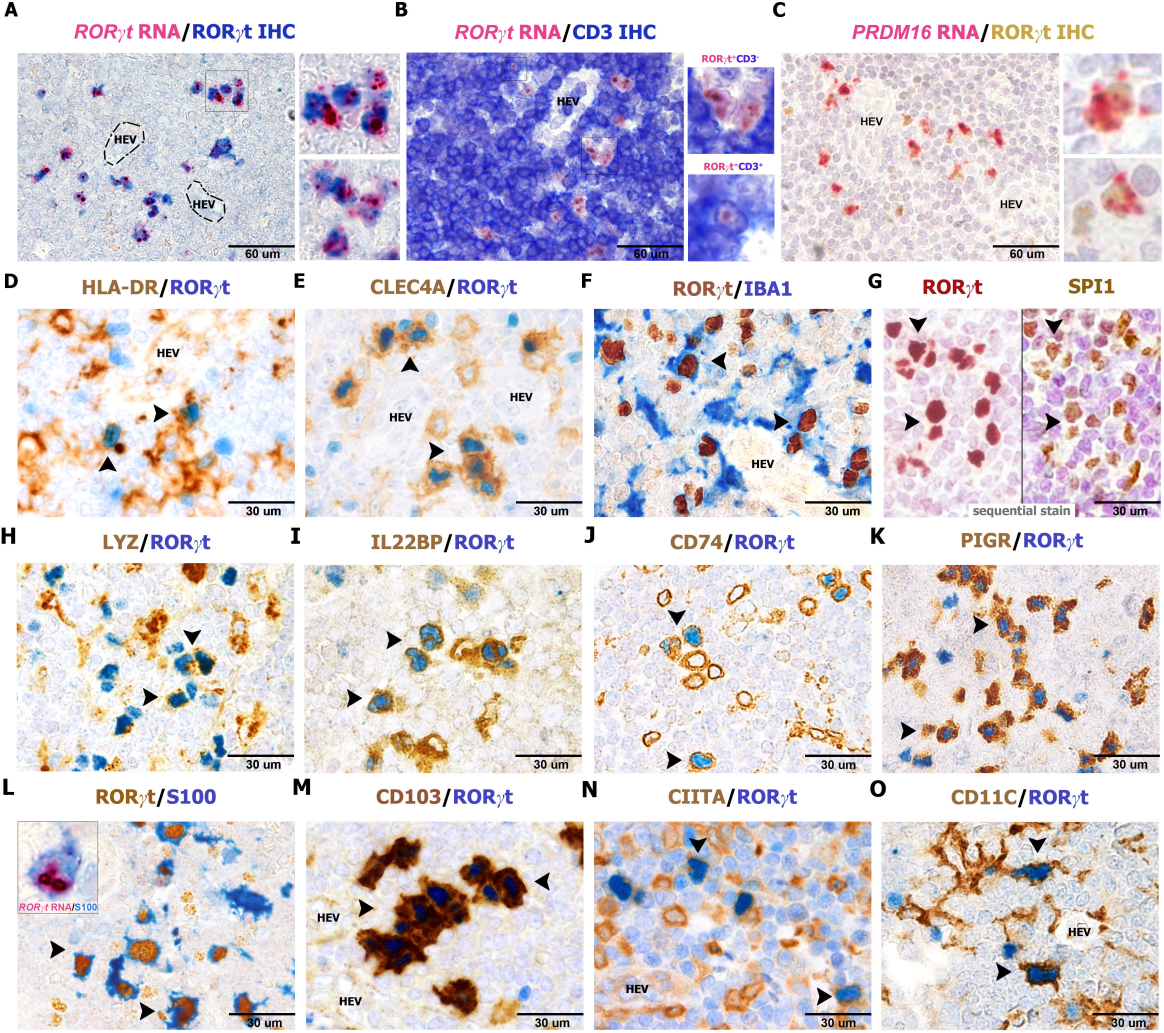
Phenotype of RORγt-DCs in lymph nodes. Sections are from human lymph nodes **(A–O)** and stained as indicated by labels. RORγt^+^CD3^−^ expresses a high amount of *RORγt* and *PRDM16* transcripts **(A–C)** by RNAscope technique. Note that RNAscope signals in **(A, B)** are more in RORγt-DCs than in RORγt^+^CD3^+^ T cells as shown in the bottom inset in panel **(B)** RORγt cells display a DC morphology and co-express (arrows) HLA-DR (**D**, n=10), CLEC4A (**E**, n=25), AIF1/IBA1 (**F**, n=6), the transcription factor SPI1 (**G**, n=4), LYZ (**H**, n=6), IL22BP (**I**, n=6), CD74 (**J**, n=5), PIGR (**K**, n=15), S100 (**L**, n=20), CD103 (**M**, n=25), CIITA (**N**, n=5), and CD11C (**O**, n=6). Transcript for *RORγt* is found in the cytoplasm of S100^+^ cells (**L**, inset) showing dendritic morphology. **(G)** Sequential immunostaining; corresponding cells bearing both markers are indicated by arrows.

**Figure 4:**
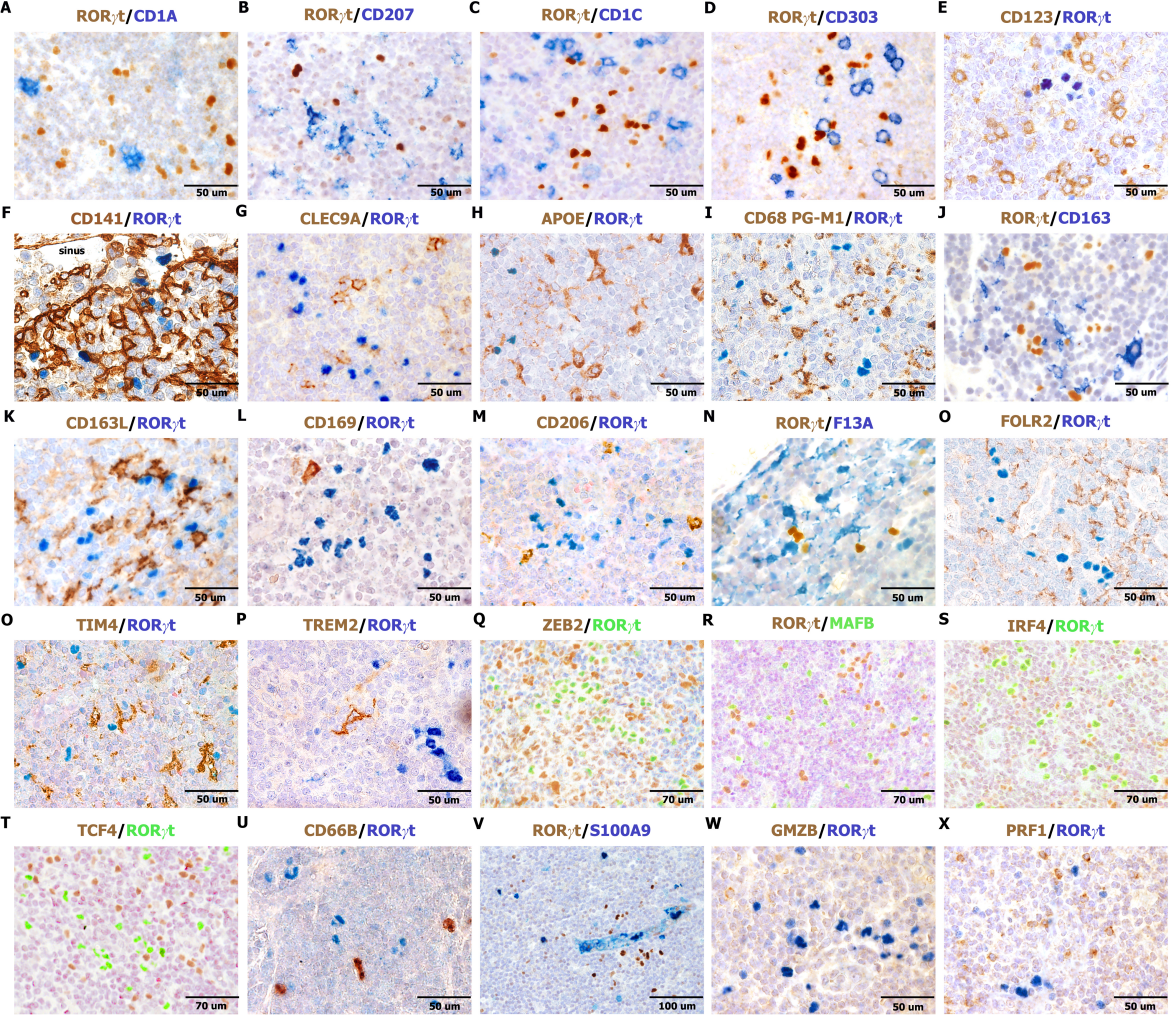
Myeloid, DC, and macrophage markers negative in RORγt-DCs in lymph node. Sections are from human reactive lymph nodes (n=3) **(A–X)** and double stained as indicated by labels. RORγt-DCs are negative for CD1A, CD207, CD1c, CD303, CD123, CD141, CLEC9A, APOE, CD68/PG-M1, CD163, CD163L, CD169, CD206, F13A, FOLR2, TIM4, TREM2, ZEB2, MAFB, IRF4, TCF4, CD66b, S100A9, GZMB, and PRF1 **(A–X)**. **(Q–T)** Images were obtained from digital slides sequentially immunostained, digitally resized and overlapped adopting artificial colors using Adobe Photoshop.

In our previous study, the characterization of RORγt-DCs found in gut lamina propria was mainly based on transcriptomic analysis (25). Among specific transcript expressed by RORγt-DCs previously identified, we could validate high levels of *RORγt* and *PRDM16* expression on lymph node sections by RNAscope (**Figures 3A–C**). We further confirmed expression of HLA-DR, CLEC4A, AIF1/IBA1, SPI1, LYZ, IL22RA/IL22BP, CD74, and the polymeric immunoglobulin receptor (PIGR) (**Figures 3D–K**). PIGR is a glycoprotein located at the basolateral side of intestinal epithelial cells that mediates the transcytosis of IgA, produced by plasma cells in the gut lamina propria. Remarkably, PIGR was highly restricted to RORγt-DCs among immune cells (**Figure 3K**). We could also detect recurrent expression of S100 protein (using an antibody that recognizes S100B, S100A1, and S100A6 isoforms), CD103, CIITA (**Figures 3L–N**), and CD11C (**Figure 3O**). RORγt-DCs did not express other additional markers expressed in macrophages and DC subsets. Specifically, they rendered negative results for many conventional DCs and Langerhans cells markers (i.e., CD1A, CD207, CD1C, CD303, CD123, CD141, CLEC9A; n=3, **Figures 4A–G**), classical macrophage markers (i.e. APOE, CD68 [PG-M1], CD163, CD163L, CD169, CD206, F13A, FOLR2, TIM4, and TREM2; **Figures 4H–P**), and transcription factors (TFs) typically found in MΦs (ZEB2 and MAFB; **Figures 4Q, R**) and DCs (IRF4 and TCF4; **Figures 4S, T**). No reactivity was found for markers of myeloid cells CD66B and S100A9 (**Figures 4U, V**) as well as the cytotoxic molecules GZMB and PRF1 (**Figures 4W, X**). We also explore the abundance of RORgt-DCs and other DCs in the interfollicular area of lymph nodes by analyzing cases (n=4) containing an enriched RORgt-DCs population (**Supplementary Table S1**). In two of them, the RORγt-DCs component was dominant and outnumbered PDCs, cDC1, and cDC2 DCs in selected areas around HEV (**Supplementary Figure S4**). All these findings confirm that RORγt-DCs found in draining lymph nodes represent a distinct DC population, as also recently proposed (37).

**Figure 5:**
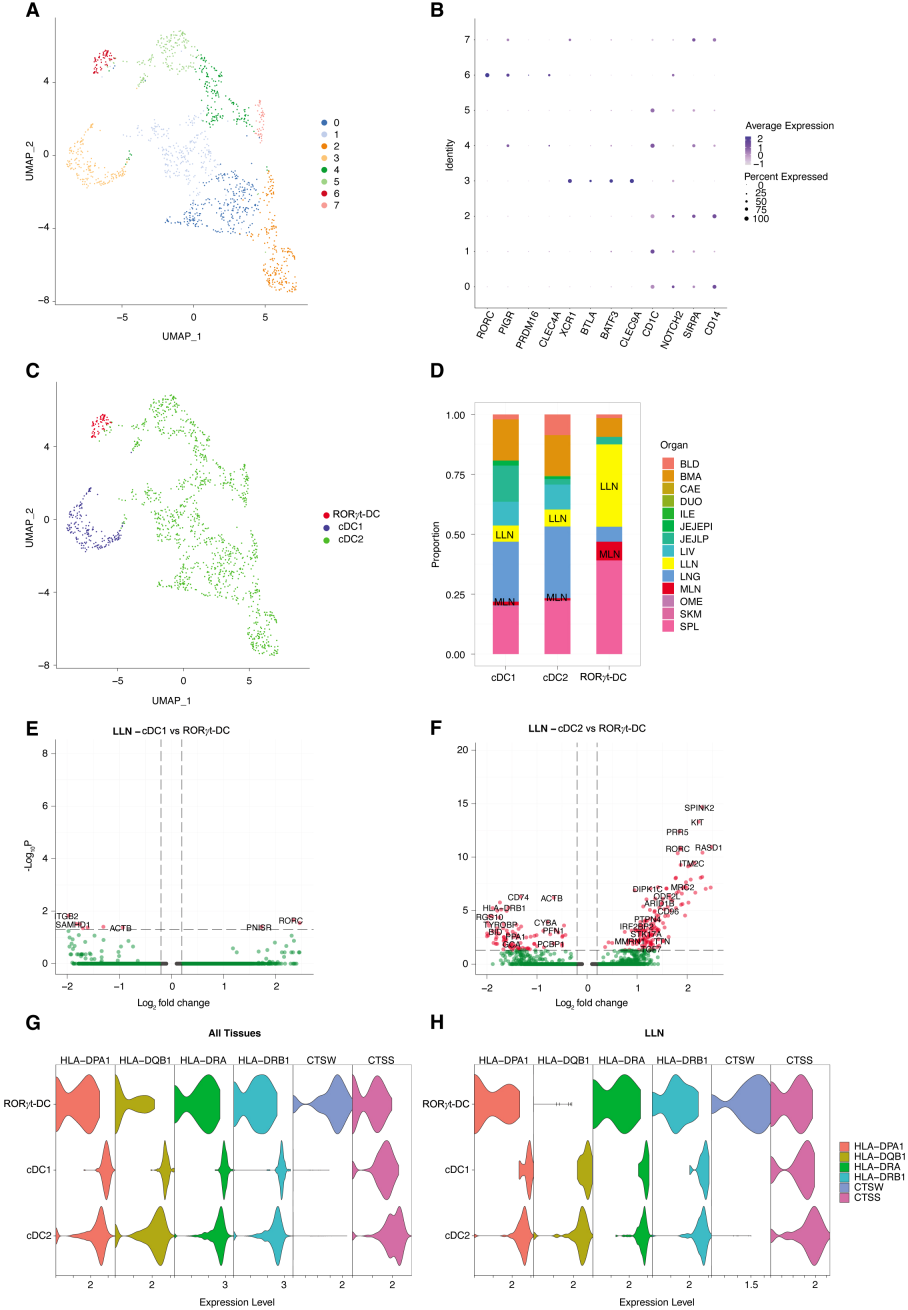
scRNAseq analysis identifies RORγt-DCs in human lymph nodes. **(A)** UMAP of cDC1; cDC2; RORγt-DCs from human Cross-tissue Immune Cell Atlas (CTICA) dataset (n=12 adult organ donors; total, 1394 cells). **(B)** Dot plot of selected cell type defining genes. **(C)** Annotated UMAP by cell type based on gene signatures in panel **(B)**. **(D)** Bar plot representing the tissue distribution of each cell type. BLD, blood; BMA, bone marrow; CAE, caecum; DUO, duodenum; ILE, ileum; JEJEPI, jejunal intraepithelial layer; JEJLP, jejunal lamina propria; LIV, liver; LLN, lung-draining lymph nodes; LNG, lung; MLN, mesenteric lymph node; OME, omentum; SCL, sigmoid colon; SKM, skeletal muscle; SPL, spleen. **(E; F)** Volcano plots showing differentially expressed genes in lung-draining lymph nodes of RORγt-DCs vs. cDC1 DCs **(E)** or vs. cDC2 DCs **(F)**. **(G, H)** Violin plots depicting expression of selected genes associated with antigen presentation mechanism across cell types in **(G)** all tissues and in **(H)** LLN.

We further expand and validate this finding by confirming their occurrence, similarity with other nodal DC populations, and antigen presenting features by analyzing human scRNAseq dataset curated in the Cross-tissue Immune Cell Atlas (CTICA) (38). This dataset includes genes of 45 well-annotated immune cell types from 16 different tissues of 12 adult organ donors, providing defined signatures of known immune cell subtypes and valuable information of their tissue distribution. To increase the resolution of our analysis, we first bioinformatically removed known cell types, such as T cells, B cells, monocytes, plasma cells, and conventional ILCs, based on cell type specific markers. We then extracted all remaining cells expressing the XCR1, CD1C, and RORC transcripts from the CTICA and recluster them on UMAP, obtaining a total of eight clusters (**Figure 5A**). Cluster 6 corresponded to RORγt-DCs, given the defining gene signature of RORC, PIGR, PRDM16, and CLEC4A (**Figures 5B, C**). Cluster 3 represented cDC1 population with the expression of XCR1, BTLA, BATF3, and CLEC9A (**Figures 5B, C**). Based on the high expression level of CD1C, NOTCH2, SIRPA, and CD14, we defined clusters 0, 1, 2, 4, 5, 7 as cDC2. Analysis of the tissue abundance of each cell type revealed that RORγt-DCs were prevalently found in spleen, lung-draining lymph nodes (LLN), and mesenteric lymph node (MLN) (**Figure 5D**). We then asked whether nodal RORγt-DCs display a specific transcriptomic profile. To this end, we selected LLN for downstream analysis, given the relative abundance of RORγt-DCs over that of MLN. Many differentially expressed genes (DEGs) were observed in RORγt-DCs compared to nodal myeloid DCs (**Figures 5E, F**); however, the comparison between RORγt-DCs and cDC1 did not yield a robust DEG profile (**Figure 5E**), likely due to the scarcity of these cells. We further examined the expression of the antigen presentation machinery on RORγt-DCs and myeloid DCs (**Figure 5G**). We observed that HLA-DR and HLA-DQ were indeed expressed in RORγt-DCs in LLN (**Figure 5H**). Together, these data identified a small subset of RORγt-DCs which are distinct from conventional nodal myeloid DCs but also express genes related to the antigen presentation machinery in human lymph nodes.

### RORγt-DCs occurrence in lymphoid and non-lymphoid human tissues

**Figure 6:**
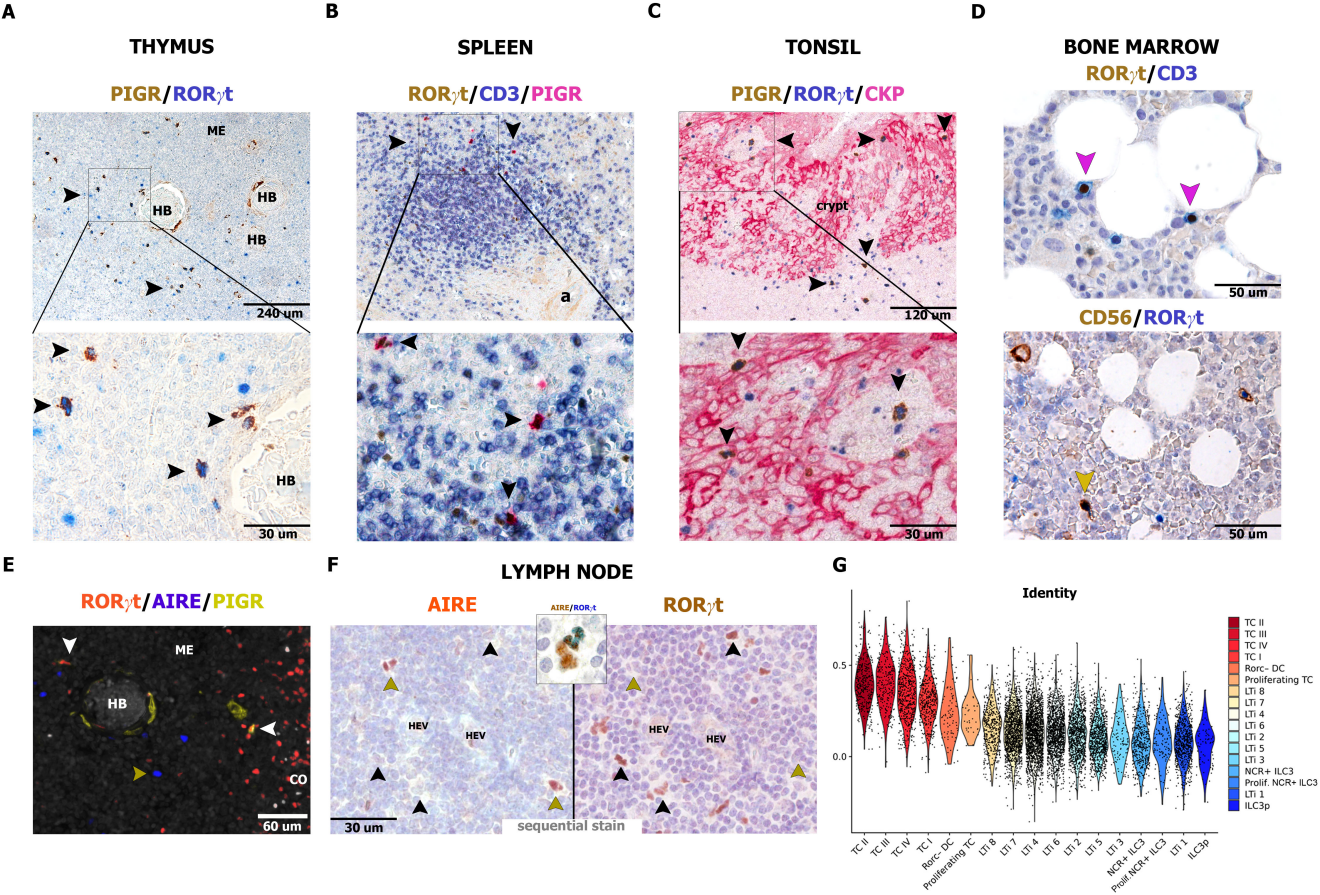
Distribution of RORγt-DCs and AIRE expression in human lymphoid organs. Sections are from human thymus **(A, E)**, spleen **(B)**, tonsil **(C)**, bone marrow, **(D)** and reactive lymph node **(F)** and stained as indicated by labels. RORγt-DCs in the thymus are found in the medulla close to Hassall bodies [HB, **(A)**] and admixed to AIRE^+^ epithelial cells (n=4, **E**). In the spleen, RORγt-DCs are limited to periarteriolar lymphoid sheets, within T-cell aggregates (**B**, n=10). In tonsils, RORγt-DCs are found mainly in the crypt epithelium as well as in the interfollicular area (**C**, n=24). In bone marrow (n=22), RORγt-DCs are lacking and reactivity for RORγt is limited to CD3^+^ T cells and CD56^+^ ILC3 **(D)**. In reactive lymph nodes, AIRE reactivity is extremely rare in RORγt-DCs as revealed by sequential immunostain (**F**, n=10, in inset example of a cell AIRE^+^ showing weak positive staining for RORγt). **(G)** Violin plot showing scaled and normalized module score enrichment of the signature of RORγt-DCs (Rorc-DC) from lamina propria (n=6 donors) from Ulezko Antonova & Lonardi et al. (25) in single cells from Akagbosu et al. (26) grouped by the authors’ cell type annotation and sorted by decreasing average enrichment. To perform the mouse-to-human comparison, all mouse genes present in the publicly available counts matrix from Akagbosu et al. were first converted into their human homologs (23459 total genes).

We subsequently analyzed in detail the presence of similar cells in peripheral lymphoid organs, such as thymus, spleen, tonsils, and bone marrow (**Figure 6**; **Supplementary Figure S5** and **Supplementary Table S3**), and non-lymphoid tissues (**Supplementary Figure S6** and **Supplementary Table S4**). RORγt-DCs were largely absent in peripheral non-lymphoid tissues (**Supplementary Figure S6** and **Supplementary Table S4**), except for scatter cells in the gut lamina propria as already described (25), confirmed in this analysis by PIGR co-expression. RORγt-DCs are regularly found in the thymic medulla (n=4; **Figure 6A**), in the T-cell area of the spleen (n=10; **Figure 6B**; **Supplementary Figure S5A**), and in the intraepithelial and interfollicular areas on tonsils (n=24; **Figure 6C**; **Supplementary Figures S5B-E**); no RORγt-DCs were observed in the bone marrow (n=22; **Figure 6D**). We also explored fetal lymph nodes and could confirm a tiny fraction of RORγt-DCs among RORγt^+^ cells (**Supplementary Figure S7**). Notably, the large majority of thymic and nodal RORγt-DCs were negative for AIRE (**Figures 6E, F**), distinguishing them from mouse TCI and III and JC. Conversely, comparison of the phenotypes of RORγt-DCs with those of TCs reveals more similarity of human RORγt-DCs with mouse TCII (**Figure 6G**). Overall, these data indicate that RORγt-DCs populate human lymphoid organs.

### RORγt-DCs are absent in peripheral blood and display proliferative activity in lymph nodes

**Figure 7:**
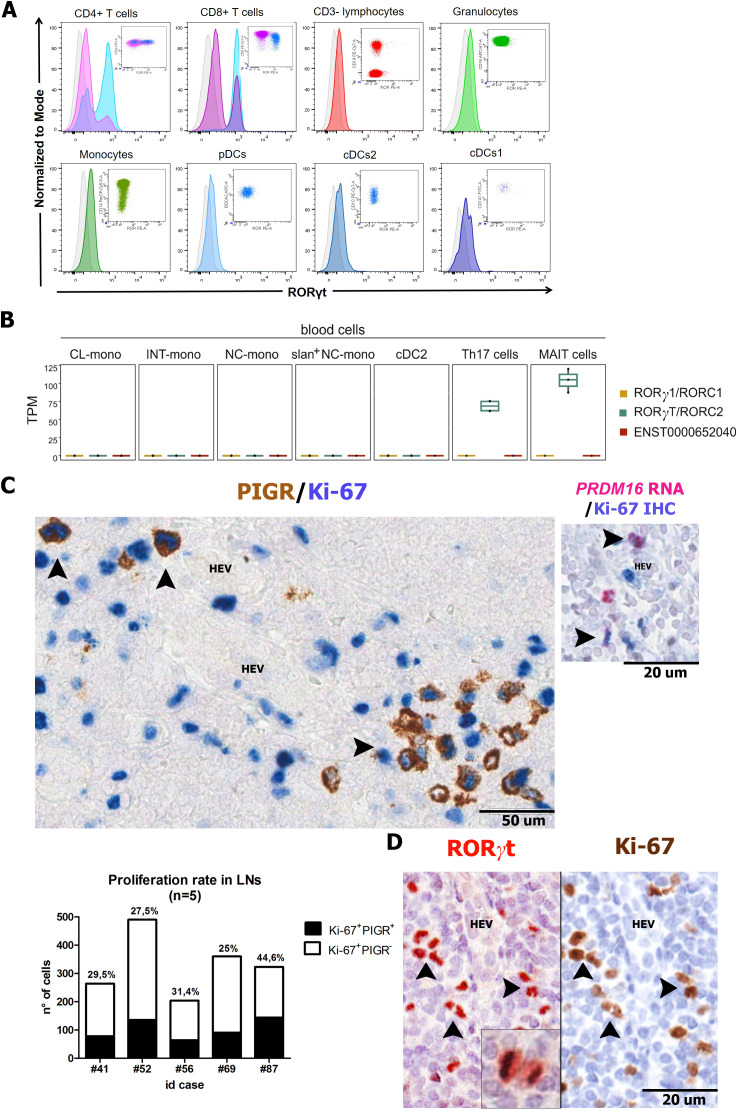
RORγt expression on peripheral blood immune cells and local renewal of nodal RORγt-DCs. **(A)** RORγt expression on peripheral blood immune cells by flow cytometry. Representative histogram and dot plot panels illustrate RORγt expression, as seen as fluorescence intensity (x-axis), in different immune cell subsets on whole blood sample. Positive cells include CD4^+^ T cells (pink) and CD45RA^−^CCR6^+^CD161^+^ Th17 cells (turquoise); CD8^+^ T cells (violet) and CD45RA^−^CCR6^+^CD161^+^ Tc17 cells (turquoise); whereas no positive staining is observed on CD3^−^ lymphocytes (red); CD16^+^ granulocytes (light green); CD14^+^/CD16^+^ monocytes (olive drab); BDCA2^+^ pDCs (light blue); CD1C^+^ cDC2s (blue) and CD141^+^ cDC1s (dark blue). Gray histogram represents FMO control signal for each population tested. Immune cell subsets are gated as described in the Materials and Methods section. **(B)** Boxplot showing the expression levels of transcriptional variants for the RORC genes in monocyte subsets (CL = classical monocytes, INT = intermediate monocytes, NC = non-classical monocytes and slan^+^ non-classical monocytes). Additionally, RORC gene expression in type-2 conventional dendritic (DC2), T-helper 17 (Th17), and Mucosal-associated invariant T (MAIT) cells is also reported in the boxplot. **(C)** Sections from a representative lymph node (n=5) and stained as indicated by labels. *PRDM16* transcript is expressed in proliferating RORγt-DCs surrounding an HEV as also illustrated by the histogram showing the absolute and relative count of proliferating Ki-67^+^PIGR^+^ cells in lymph nodes (n=5, details in **Supplementary Table S1**, mean 31,6%). **(D)** Sequential immunostainings of lymph node sections (n=3) show numerous RORγt^+^ cells surrounding high endothelial venules (HEV) expressing the proliferation marker Ki-67. Double positive cells are indicated by arrows. A RORγt positive cell in anaphase is in the inset.

To shed light on the origin of tissue RORγt-DCs, we tested the peripheral blood compartment. By exploring the scRNAseq dataset of peripheral blood mononuclear cells, we could not detect any cluster corresponding to RORγt-DCs (25). We further explored this finding by using flow cytometry on whole blood obtained from healthy donors (n=3). Among CD3^+^ T cells, the CD4^+^Th17 and CD8^+^ Tc17 subsets, identified by their expression of CCR6 and CD161, were positive for RORγt, whereas no reactivity was detected on CD3^−^ mononuclear cells and CD16^+^ granulocytes (**Figure 7A**). Among mononuclear cells, RORγt protein expression was negative on monocytes subsets (based on CD14 and CD16 expression) as well as on BDCA2^+^ plasmacytoid DCs, CD141^+^ cDC1, and CD1C^+^ cDC2 (**Figure 7A**). In keeping with the immunostain results, *RORγt/RORC2* variant transcript could not be identified in sorted purified blood monocytes and blood DC populations (**Figure 7B**). Emerging findings indicate that also the DC and MΦ compartments are maintained by the local proliferation of their progenitor or differentiated counterparts (39–41). Double staining for Ki-67 and PIGR and Ki-67 and *PRDM16* (**Figure 7C**) as well as stains for Ki-67 and RORγt on sequential lymph node sections (**Figure 7D**) suggested that a significant fraction (mean 31.6%; n=5) of nodal RORγt-DCs in the interfollicular area enter the cell cycle (**Figures 7C, D**). All these findings indicate that RORγt-DCs are distinct from other well-recognized human DC populations and are likely maintained by local renewal.

## Discussion

Dendritic cells (DCs) are key to pathogen detection and antigen presentation, shaping adaptive immune responses (18). They are highly heterogeneous, with distinct subtypes identified by lineage, localization, and function (19, 20). Upon activation, DCs mature and migrate to lymphoid organs to prime and regulate T-cell activation. Recent studies have proposed the existence of novel populations of murine RORγt-expressing cells with antigen presenting functions and variably referred to as TC or JC (20, 24, 26, 42–44). We have also recently unveiled human RORγt-DCs mainly in the gut displaying a relevant antigen presenting machinery (25). Reanalysis of published scRNAseq datasets has resulted in a reconciliation of human and mouse data on RORγt-expressing cells by confirming a substantial transcriptional overlap (45). By optimizing reagents for the identification of RORγt mRNA and protein, this study expands the characterization of human RORγt^+^ cells. Specifically, we identified RORγt-DCs in most lymphoid organs, particularly in reactive lymph nodes and tonsils. A significant fraction of nodal RORγt-DCs expressed the proliferation marker Ki-67, suggesting local self-renewal. In mouse, RORγt-expressing antigen presenting cells include TC subsets and JC (26, 42), which are capable of inducing microbiota-dependent peripheral Treg differentiation. Two out of four (TCI and TCIII) subsets and JC express the autoimmune regulator *AIRE* mRNA and protein together with a transcriptional overlap with AIRE-expressing medullary thymic epithelial cells. The present study indicates that the large majority of human RORγt-DCs in adult tissues lack AIRE expression. In fact, they overlap with TCII.

Although the function of RORγt-DCs remains under active investigation, a set of experimental findings on human and mouse RORγt-DCs suggests that they can process and present antigens, differentiate to cDC2, migrate to lymph nodes, and present antigens to naïve CD4^+^ T cells. These findings are consistent with data from the present study on nodal RORγt-DCs, which confirm their dendritic cell-like morphology and antigen-presenting machinery at both the transcriptional and protein levels. Notably, we observed that RORγt-DCs are primarily localized to lymphoid organs but absent in many non-lymphoid tissues. Moreover, they display a high proliferative activity. These observations align with the hypothesis that their pool and subsequent differentiation are sustained and expanded by local signals, which help organize nodal immune responses. However, like other immune cell types, RORγt-DCs may also localize to and proliferate in inflamed non-lymphoid tissues, as well as recirculate through the lymph in various pathological conditions.

Overall, this study offers both phenotypic and molecular evidence supporting the identification of the novel human RORγt-DC population. Their mapping in human tissues through microscopy will aid in better defining their role in various pathologies. It is important to note that these cells are relatively rare, which poses challenges in studying them in detail. As with other rare cell types, refining expansion protocols will be crucial for advancing our understanding of their biology and function, particularly with respect to the human counterpart.

## Data Availability

Publicly available datasets were analyzed in this study. This data can be found here: http://www.ncbi.nlm.nih.gov/geo/GEO GSE107011 and GSE136107.
