# Supplementary material for: Mapping of RORγt+ dendritic cells in human tissues establishes their preferential niche in adult lymph nodes
Source: Front Immunol. 2025 May 30;16:1527499. doi: 10.3389/fimmu.2025.1527499 (PMC12162286; doi:10.3389/fimmu.2025.1527499)
Supplement: Supplementary file 1 [file DataSheet1.pdf]

Supplementary Tables

Supplemental Table 1. List of lymph nodes analyzed for RORγt-DC content (semi- quantitative scoring) separated for diagnosis.

DIAGNOSIS	*ID*	*SITE*	*AGE*	*GENDER*	*Area LN (mm^2)*	*Score*
DERMATOPATHIC LYMPHADENITIS						
	1	Superficial lymph node	*0*	F	47,4	+
	2	Mediastinal	*1*	M	17	+
	3	Inguinal	2	F	50,3	+
	4	Inguinal	41	F	61,2	+
	5	Axillary	47	M	45,7	+
	6	Axillary	49	M	33,8	+
	7	Inguinal	53	M	88,4	+
	8	Inguinal	53	M	48,5	+
	9	Inguinal	62	M	58,5	++
	10	Axillary	66	M	47	++
	11	Inguinal	66	M	131,7	+
FOLLICULAR HYPERPLASIA						
	12	Upper deep cervical (level II)	1	M	68,8	++
	13	Upper deep cervical (level IIB)	1	M	20,5	+
	14	Lateral cervical	3	F	49,8	+
	15	Lateral cervical	4	M	316,5	+
	16	Lateral cervical	5	M	36	++
	17	Mediastinal	5	M	40,8	+
	18	Appendix	6	F	14	+
	19[#]	Inguinal	7	M	175,7	+++

	20	Colon	8	F	6,4	+
	21	Axillary	12	M	322,1	+++
	22	Epitrochlear	13	M	140,1	+
	23	Axillary	13	M	23,7	+
	24	Mesenteric	18	F	145,2	+++
	25	Lateral cervical	19	F	87,5	++
	26	Upper and middle deep cervical (level II-III)	19	M	109,8	+++
	27	Retroauricular	25	M	55,8	+
	28	Axillary	28	F	50,6	+++
	29	Axillary	28	M	14,8	+
	30[#]*	Submental (level Ia)	29	F	195,1	+++
	31	Inguinal-femoral	32	F	67,6	+
	32	Upper deep cervical (level IIa)	33	M	285	+++
	33	Pectoral	36	M	66,7	+++
	34	Axillary	37	F	25	+
	35	Lateral cervical	40	M	92,5	+
	36	Mediastinal	40	F	67,1	+
	37	Supraclavicular	40	M	23,5	+
	38	Axillary	41	M	61,1	++
	39	Neck (level II)	41	F	67,6	+
	40[$]	Axillary	41	F	45,2	+
	*41**	Inguinal-femoral	42	M	132,9	++
	42	Upper deep cervical (level IIa)	42	M	227,1	+++
	43	Mesenteric	45	F	42,6	+
	44	Inguinal	45	M	99,6	++
	45[#]*	Posterior cervical	47	F	116,9	+++
	46	Submandibular	50	F	28,1	+++
	47	Axillary	52	F	39,1	+
	48	Inguinal	53	F	41	+
	49	Parotid	54	M	87,2	+
	50	Submental (level I)	55	M	111,6	+++

	51	Supraclavicular	56	F	10,9	+
	52	Axillary	57	F	38,4	+
	53	Lateral cervical	58	F	44,7	+
	54	Lateral cervical	58	M	84,5	+++
	55	Ileum	59	M	4,9	++
	*56**	Inferior parathyroid pole	63	F	156,6	+++
	57	Lateral cervical	64	M	46,7	+
	58	Inguinal	64	M	94,2	+++
	59	Axillary	66	M		+++
	60	Submandibular (level Ib)	67	M	97,9	++
	61	Supraclavicular	68	F	27,8	++
	62	Lateral cervical	70	F	39,7	+
	63	Lateral cervical	71	F	20,5	+
	64	Hilar	71	M	102,2	++
	65	Lateral cervical	72	M	37,6	++
	66	Axillary	74	M	90,8	+++
	67	Pectoral	75	F	160,2	+
	68	Submandibular	76	F	82,7	+
	69	Iliac	77	M	54,5	+
	70[$]	Axillary	77	M	24,9	+
	71	Lateral nuchal	77	F	7	+
	72	Iliac	79	F	7	+
PROGRESSIVELY TRANSFORMED GERMINAL CENTERS						
	73	Lateral cervical (level V)	*8*	M	136,5	+
	74[#]	Axillary	17	M	96,9	+++
	75	Axillary	17	M	148,8	++
	76	Cervical	18	M	137,3	+
	77	Inguinal	19	M	119,7	+++
	78[$]	Upper deep cervical (level IIa)	22	M	90,8	+++
	79	Submental	36	M	79,5	+
	80	Axillary	49	F	216,9	+
	*81**	Axillary	52	F	103,3	+++
	82	Supraclavicular	56	F	143,2	+
	83	Axillary	57	F	191,7	+
	84	Lateral cervical	57	M	88,6	+++
	85	Submental	59	F	36,9	++
CASTLEMAN DISEASE						

	86	Hilar	38	F	317,5	+
	**87*	Supraclavicular	*40*	F	32,2	+++
	88	Inferior jugular	59	F	53,9	+
GRANULOMATOUS LYMPHADENITIS						
	89	Lateral cervical (level *III)*	*0*	M	*8,7*	+
	90	Lateral cervical	4	F	65,3	+
	91°	Supraclavicular	44	M	99,8	+
	92[$]	Axillary	53	F	71,5	+++
	93	Axillary	66	M	69,3	+
		SARCOIDOSIS				
	94	Supraclavicular	41	M	112,2	+
	95	Lateral cervical	43	F	147,2	+
NECROTISING GRANULOMATOUS LYMPHADENITIS						
	96	Paratracheal	*2*	M	245,7	+
	97	Submandibular	4	M	86,3	+
		**MYCOBACTERIAL**				
	98	Lateral cervical	2	F	208,4	+
	99	Hepatic	2	F	102,2	+
	100	Cervical	5	M	408,3	+
	101	Submandibular	8	F	12	+
	102	Supraclavicular	36	F	161,7	+
	103	Superficial lymph node	37	F	52,1	+
	104	Lateral cervical	46	F	20,1	+

Legend: * = Cases shown in Figure 2F and Figure 6C. [#] = Cases shown in Supplementary Figure 5. ° = Case shown in Figure 1F. [$] = Cases used for identification of ROR□t^{+}CD3^{-}CD127^{-} cells by flow citometry shown in Figure 2G.

Supplementary Table 2

Patients' and samples' characteristics. P-value estimated by Fisher's exact test # or Kruskal–Wallis test§.

	**Overall N=104**	**-/+ N=66 (63.5%)**	**++ N=15 (14.4%)**	**+++ N=23 (22.1%)**	**P-value**
**Gender**					
M	56 (53.8%)	29 (43.9%)	12 (80.0%)	15 (65.2%)	<0.001 #
F	48 (46.2%)	37 (56.1%)	3 (20.0%)	8 (34.8%)	
**Age**					
Mean (SD)	38.9 (23.9)	37.0 (25.1)	46.3 (24.7)	39.5 (19.6)	0.44§
Median [Min, Max]	41.0 [0, 79.0]	41.0 [0, 79.0]	59.0 [1.00, 72.0]	40.0 [7.00, 74.0]	
**Site**					
Abdomen	12 (11.5%)	9 (13.6%)	2 (13.3%)	1 (4.3%)	0.553#
Axillary	22 (21.2%)	13 (19.7%)	3 (20.0%)	6 (26.1%)	
Cervical	51 (49.0%)	32 (48.5%)	7 (46.7%)	12 (52.2%)	
Inguinal	13 (12.5%)	7 (10.6%)	3 (20.0%)	3 (13.0%)	
Mediastinal	3 (2.9%)	3 (4.5%)	0 (0%)	0 (0%)	
Superficial other site	3 (2.9%)	2 (3.0%)	0 (0%)	1 (4.3%)	
**Depth**					
Deep node	15 (14.4%)	12 (18.2%)	2 (13.3%)	1 (4.3%)	0.275#
Superficial node	89 (85.6%)	54 (81.8%)	13 (86.7%)	22 (95.7%)	
**Node area**					

	Overall N=104	-/+ N=66 (63.5%)	++ N=15 (14.4%)	+++ N=23 (22.1%)	P-value
Mean (SD)	91.1 (76.7)	83.2 (80.5)	69.8 (40.7)	128 (73.5)	0.0761 §
Median [Min, Max]	68.2 [4.90, 408]	54.2 [6.40, 408]	61.1 [4.90, 149]	110 [28.1, 322]	
**Diagnosis**					
Castelman disease	3 (2.9%)	2 (3.0%)	0 (0%)	1 (4.3%)	0.203#
Dermatopathic lymphadenitis	11 (10.6%)	9 (13.6%)	2 (13.3%)	0 (0%)	
Follicular hyperplasia	61 (58.7%)	33 (50.0%)	11 (73.3%)	17 (73.9%)	
Granulomatous lymphadenitis	16 (15.4%)	16 (24.2%)	0 (0%)	0 (0%)	
Progressively transformed germinal centers	13 (12.5%)	6 (9.1%)	2 (13.3%)	5 (21.7%)	

Supplemental Table 3. Primary and secondary lymphoid tissue (without lymph nodes) analyzed for RORγt-DC content.

SITE	*ID*	*AGE*	*GENDER*	*DIAGNOSIS*
BONE MARROW n=22				
	1	46	M	reactive
	2	47	F	reactive
	3	67	F	reactive
	4	70	M	reactive
	5	87	M	reactive
	6	87	F	reactive
	7◆	50	F	reactive
	8◆	64	M	reactive
	9◆	48	F	reactive
	10◆	85	M	reactive
	11#	61	M	reactive
	12*	29	M	reactive
	13*	45	M	reactive
	14*	59	F	reactive
	15*	60	M	reactive
	16*	66	F	reactive
	17*	69	F	reactive
	18*	70	F	reactive
	19*	70	F	reactive
	20*	75	F	reactive
	21*	75	F	reactive
	22*	79	F	reactive
SPLEEN n=10				
	23	49	M	extramedullary hematopoiesis
	24	34	F	reactive
	25	44	M	reactive
	26	15	M	reactive
	27▽	18	M	reactive
	28▽	18	M	reactive
	29▽	24	F	reactive
	30	43	M	reactive
	31	52	M	reactive
	32▽	57	M	reactive
THYMUS n=4				

	29%	3	M	reactive
	30	23	F	reactive
	31	29	F	reactive
	32$	50	F	reactive
TONSIL n=24				
	33	2	M	reactive
	34	3	M	reactive
	35	3	M	reactive
	36	3	M	reactive
	37	4	F	reactive
	38	4	M	reactive
	39	4	F	reactive
	40	5	M	reactive
	41	5	F	reactive
	42	5	F	reactive
	43	5	M	reactive
	44	5	M	reactive
	45	6	F	reactive
	46	6	F	reactive
	47	7	M	reactive
	48	8	M	reactive
	49	8	F	reactive
	50	9	M	reactive
	51	9	F	reactive
	52	9	F	reactive
	53	12	M	reactive
	54	13	M	reactive
	55	15	F	reactive
	56	34	M	reactive

Legend: ♦ =evaluation after lymphoma therapy; # evaluation after myeloma therapy; *= stadiation of lymphoma; ∇= traumatic; %= free margin of cystic teratoma; $= free margin of thymoma.

Supplemental Table 4

Cases of reactive non-lymphoid tissues analyzed for RORγt-DCs.

ORGAN	AGE	GENDER	RORγT-DCs
Adrenal Gland	44	M	-
Bladder	41	M	-
Brain	28	M	-
	37	M	-
	30	F	-
Breast	30	F	-
	29	F	-
	32	F	-
	31	F	-
	34	F	-
Colon /Rectum	67	M	-
	84	M	+
	12	M	-
Duodenum/ Ileum	68	F	+
	80	F	+
	0	F	+
	59	M	+
Kidney	75	M	-
Liver	42	F	-
	65	F	-
	49	F	-
	64	F	-
	54	F	-
Lung	19	F	-
	19	F	-
	21	M	-
	20	F	-
	28	M	-
Ovarian Tube	55	F	-

Ovary	45	F	-
Pancreas	65	F	-
Placenta	34	F	-
	33	F	-
	28	F	-
Prostate	82	M	-
Salivary gland	69	M	-
Skin	25	M	-
	56	M	-
	33	M	-
	74	M	-
	25	M	-
Skeletal muscle	81	M	-
Stomach	60	F	-
	70	M	-
	59	M	-
Testis	42	M	-
Thyroid	67	F	-
Ureter	66	M	-
Uterus	70	F	-
	36	F	-
	45	F	-

Legend: += presence of scattered RORγt-DCs; - = absence of RORγt-DCs.

Supplemental Table 5

Fetal lymph nodes from autopsy stained for RORγt, PIGR, CLEC4A, CD3, CD127 and CD117.

ID	*WEEKS+DAYS*	*GENDER*	*DIAGNOSIS*	*NUMBER OF LYMPH NODES*
1	18	M	congenital hernia	2
2	19+5	F	Wolf-Hirschhorn Syndrome	1
3	20+5	F	congenital deformation	25
4	20	M	congenital malformation of the nervous system	7
5	21+6	M	agenesis	2
6	22	M	DiGeorge Syndrome	1
7	22	F	malformation of Dandy Walker	3

Supplementary Table 6.
List of the antibodies used for IHC

Reagent	Clone	Dilution	RRID	Source
**PRIMARY ANTIBODIES**				
AIF-1/IBA1	E404W	1:1800	AB_2820254	Cell Signaling Technology
AIRE	Goat pAb	1:900	AB_1565824	Abcam
APOE	EP1374Y	1:500	AB_867704	Abcam
CD1a	010	1:50	AB_2073290	Agilent Technologies
CD1c	OTI2F4	1:300	AB_2889187	Abcam
CD3	SP7	1:100	AB_2244302	Thermo Scientific
CD4	MT310	1:50	AB_2075537	Agilent Technologies
CD8	C8/144B	1:60	AB_2075537	Agilent Technologies
CD11c	5D11	1:100	AB_10555570	Leica Biosystems
CD14	7	1:50	AB_563495	Leica Biosystems
CD16	2H7	1:100	AB_563580	Leica Biosystems
CD20	L26	1:250	AB_2282030	Agilent Technologies
CD31	JC70A	1:50	AB_2935723	Leica Biosystems
CD34	QBEnd/10	1:200	AB_61379	Thermo Scientific
CD45, LCA	2B11 + PD7/26	1:200	AB_2314143	Agilent Technologies
CD56	MRQ-42	undiluted	AB_2941091	Roche
CD66b	G10F5	1:200	AB_314494	BioLegend
CD68	PGM1	1:50	AB_2074844	Dako
CD103	EPR4166 (2)	1:500	AB_10861462	Abcam
CD117	Rb pAb	1:100	AB_2335702	Agilent Technologies
CD123	7G3	1:50	AB_395999	BD Biosciences
CD127	EPR23747-333	1:500	AB_3065027	Abcam
CD141/Thrombomodulin	EPR4051	1:60	AB_3106948	Abcam
CD163	10D6	1:50	AB_64139	Thermo Scientific
CD163L	Rb pAb	1:500	AB_2072575	Sigma-Aldrich
CD169	HSn 7D2	1:50	AB_526814	Novus Biologicals
CD206	Rb pAb	1:3000	AB_10896526	Abcam
CD207/Langerin	12D6	1:200	AB_2336548	Vector Laboratories
CD303/BDCA2	124B3.13	1:75	AB_1149764	Dendritics
CIITA	7-1H	1:50	AB_627261	Santa Cruz Biotechnology
CLA/Heca 452	HECA-452	1:50	AB_2185262	BD Biosciences
CLEC4A	G9	1:350	AB_10988022	Santa Cruz Biotechnology

CLEC9A	Sheep pAb	1:100	AB_10888664	R&D Systems
CXCL12/SDF-1	79018	1:50	AB_2088149	R&D Systems
CXCL13/BCA-1	53610	1:100	AB_2086049	R&D Systems
Factor XIIIa	AC1-A1	1:100	AB_63782	Thermo Scientific
FOLR2	OTI4G6	1:100	AB_2723188	Thermo Scientific
Granzyme B	11F1	1:20	AB_10554441	Leica Biosystgems
Keratin (wide spectrum-CKP)	MNF116	1:100	AB_2858276	Agilent Technologies
KI67	MIB-1	1:100	AB_2142367	Agilent Technologies
HLA-DR	PdV5.2	1:400	AB_2012533	Santa Cruz Biotechnology
IL22BP	EPRR23006-70	1:500	AB_3106949	Abcam
IRF4/MUM1	MUM1p	undiluted	AB_2127157	Agilent Technologies
Isotype control Mouse IgG2a	-	1:60000	-	Invitrogen
Lysozyme	Rb pAb	1:1200	AB_2341230	Agilent Technologies
MAFB	Rb pAb	1:500	AB_2799192	Merk
Multi-Cytokeratin	AE1/AE3	1:50	AB_564122	Leica
PDPN/Podoplanin	D2-40	1:40	AB_620128	Bio-Rad
PAX5	24/Pax-5	1:80	AB_398182	BD Biosciences
Perforin	5B10	1:50	AB_563955	Leica
Polymeric ig receptor/PIGR	-	1:400	AB_10677612	Abcam
PU.1/SPI1	G148-74	1:60	AB_395335	BD Biosciences
ROR gamma T	6F3.1	1:1500	AB_11205416	Merk
S100 protein	Rb pAb	1:3000	AB_10013383	Agilent Technologies
S100A9	Rb pAb	1:2500	AB_1856537	Sigma-Aldrich
SMA	1A4	1:300	AB_64001	Thermo Scientific
TCF-4/E2-2	NCI-R159-6	1:500	AB_2714172	Abcam
TCR beta	8A3	1:100	AB_2943587	Thermo Scientific
TCR delta	H-41	1:150	AB_1130061	Santa Cruz Biotechnology
TIM4	Goat pAb	1:60	AB_2240431	R&D Systems
TREM2	D8I4C	1:100	AB_2721119	Cell Signaling Technology
ZEB2	Rb pAb	1:100	AB_10603840	Sigma- Aldrich

Supplementary Table 7

List of antibodies used for flow cytometry analysis.

PERIPHERAL BLOOD SAMPLES						
**Panel #1 Identification of T cell subsets**	**Clone**	**Isotype**	**Conjugation**	**Source**	**Catalog number**	**RRID**
CCR6	G034E3	IgG2b	BV421	BioLegend	353407	AB_10916530
CD161	HP-3G10	IgG1	BV510	BioLegend	339921	AB_2562933
CD45RA	HI100	IgG2b	FITC	Becton Dickson	555488	AB_395879
CD3	UCHT1	IgG1	PerCP Cy5.5	BioLegend	300429	AB_893301
CD8	REA734	REA	PE Vio770	Miltenyi Biotec	130-110-680	AB_2659245
CD4	RPA-T4	IgG1	APC	Becton Dickson	555349	AB_398593
CD45	2D1	IgG1	APC H7	Becton Dickson	560178	AB_1645479
**Panel #2 Identification of DC subsets**	**Clone**	**Isotype**	**Conjugation**	**Source**	**Catalog number**	**RRID**
HLA-DR	REA805	REA	VioBlue	Miltenyi Biotec	130-111-947	AB_2652161
CD45	2D1	IgG1	V500	Becton Dickson	655873	AB_2870390
CD141/BDCA-3	REA674	REA	FITC	Miltenyi Biotec	130-110-358	AB_2655108
CD3	UCHT1	IgG1	PerCP Cy5.5	BioLegend	300429	AB_893301
CD14	63D3	IgG1	PerCP Cy5.5	BioLegend	367109	AB_2566711
CD16	3G8	IgG1	PerCP Cy5.5	BioLegend	302027	AB_893263
CD19	HIB19	IgG1	PerCP Cy5.5	BioLegend	302229	AB_2275547
CD1c/BDCA-1	REA694	REA	PE Vio770	Miltenyi Biotec	130-110-596	AB_2656041
CD303/BDCA-2	AC144	IgG1	APC	Miltenyi Biotec	130-097-931	AB_2661168
CD4	RPA-T4	IgG1	APC H7	Becton Dickson	560158	AB_1645478
**Panel #3 Identification of monocytes and granulocytes**	**Clone**	**Isotype**	**Conjugation**	**Source**	**Catalog number**	**RRID**
HLA-DR	REA805	REA	VioBlue	Miltenyi Biotec	130-111-947	AB_2652161
CD45	2D1	IgG1	V500	Becton Dickson	655873	AB_2870390
CD3	REA613	REA	FITC	Miltenyi Biotec	130-113-138	AB_2725966
CD15	MMA	IgM	FITC	Becton Dickson	332778	AB_2868627
CD14	63D3	IgG1	PerCP Cy5.5	BioLegend	367109	AB_2566711
CD19	SJ25C1	IgG1	PE Cy7	Becton Dickson	341113	AB_2868769
CD4	RPA-T4	IgG1	APC	Becton Dickson	555349	AB_398593
CD16	3G8	IgG1	APC H7	Becton Dickson	560195	AB_1645466
**LYMPH NODE CELL SUSPENSIONS**						
**Panel #4 Identification of Th17 subset**	**Clone**	**Isotype**	**Conjugation**	**Source**	**Catalog number**	**RRID**
CCR6	G034E3	IgG2b	BV421	BioLegend	353407	AB_10916530
CD161	HP-3G10	IgG1	BV510	BioLegend	339921	AB_2562933
CD45RA	HI100	IgG2b	FITC	Becton Dickson	555488	AB_395879
CD3	UCHT1	IgG1	PerCP Cy5.5	BioLegend	300429	AB_893301
CD4	REA623	REA	PE Vio770	Miltenyi Biotec	130-113-227	AB_2726038
CD45	2D1	IgG1	APC H7	Becton Dickson	560178	AB_1645479
**Panel #6 Identification of ILC3 and R-DC-like**	**Clone**	**Isotype**	**Conjugation**	**Source**	**Catalog number**	**RRID**
HLA-DR	REA805	REA	VioBlue	Miltenyi Biotec	130-111-947	AB_2652161
CD56	NCAM16	IgG2b	BV510	Becton Dickson	562751	AB_2732054
CD3	REA613	REA	FITC	Miltenyi Biotec	130-113-138	AB_2725966
CD14	MφP9	IgG2b	FITC	Becton Dickson	345784	AB_2868810
CD16	REA423	REA	FITC	Miltenyi Biotec	130-113-954	AB_2726427
CD19	LT19	IgG1	FITC	Miltenyi Biotec	130-091-328	AB_244222
CD127	HIL7RM21	IgG1	PerCP Cy5.5	Becton Dickson	560551	AB_1645548
CD117	104D2	IgG1	PE Cy7	Becton Dickson	339217	AB_2868720
CD45	2D1	IgG1	APC H7	Becton Dickson	560178	AB_1645479
