# Supplementary material for: Mapping of RORγt+ dendritic cells in human tissues establishes their preferential niche in adult lymph nodes
Source: Front Immunol. 2025 May 30;16:1527499. doi: 10.3389/fimmu.2025.1527499 (PMC12162286; doi:10.3389/fimmu.2025.1527499)
Supplement: Supplementary file 2 [file DataSheet2.pdf]

## Supplementary Figures

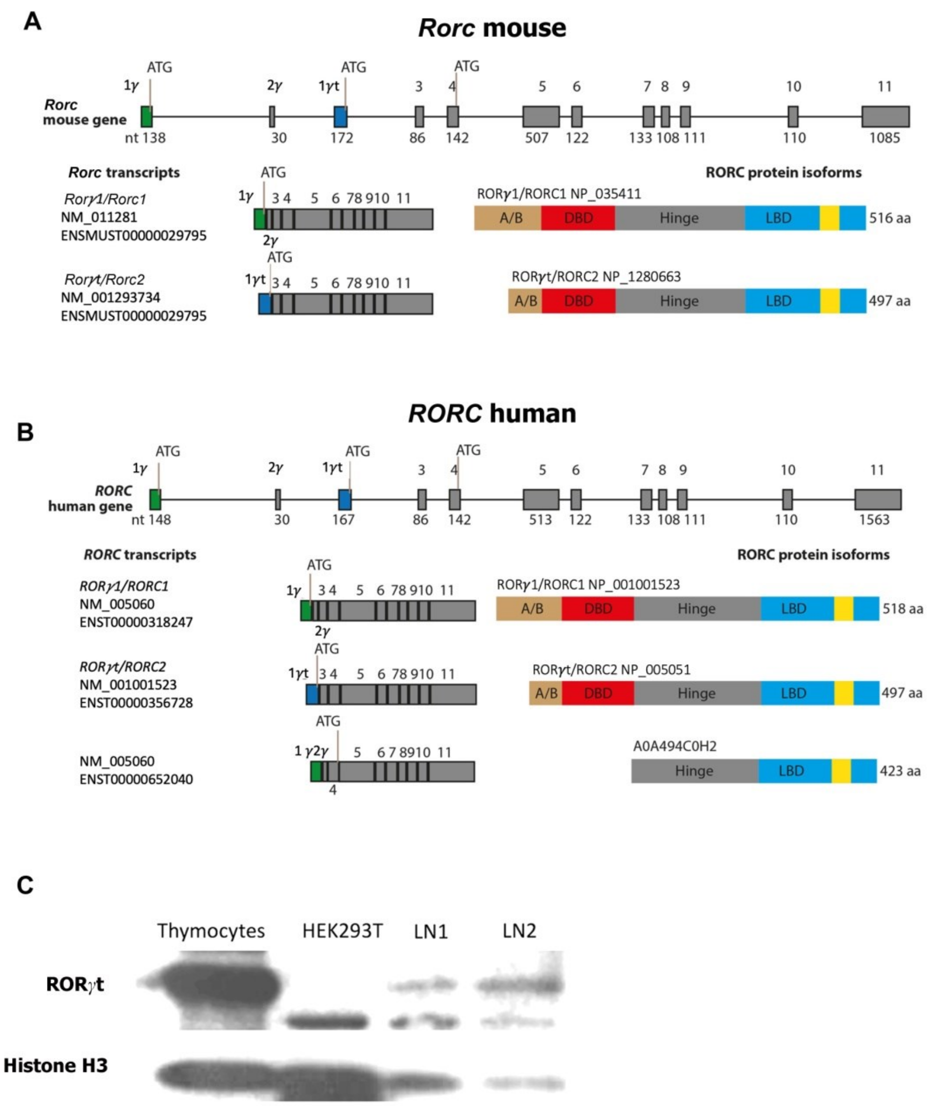


## Supplementary Figure 1.

RORC gene, transcripts and RORγt protein isoform. (**A**) Schematic representation of the mouse RORC gene. Boxes represent exons numbered 1 to 11 with their length in nucleotides (nt), lanes introns. The mRNA transcripts with the encoded proteins on the right are also shown. The usage of exon 1γ (green) and exon 2γ generates isoform RORγ1; exon 1γt (in blue) provides an alternative promoter, giving rise to RORC2/RORγt variant. The two isoforms differ in the N-terminal A/B domain structure, whereas other domains remain unchanged: DNA-binding domain (DBD), the hinge region and the ligand-binding domain (LBD) containing the activation function 2 (AF2) in yellow. (**B**) Genomic structure of human RORC gene. Skipping of exon 3 generates an additional isoform, not present in mice (NM_005060/A0494C0H2) lacking A/B and DBD domains. (**C**) immunoblots showing RORγt expression in thymocytes cell suspension, HEK293T, and lymph node cell suspensions (n=2); histone H3 was used as housekeeping control.

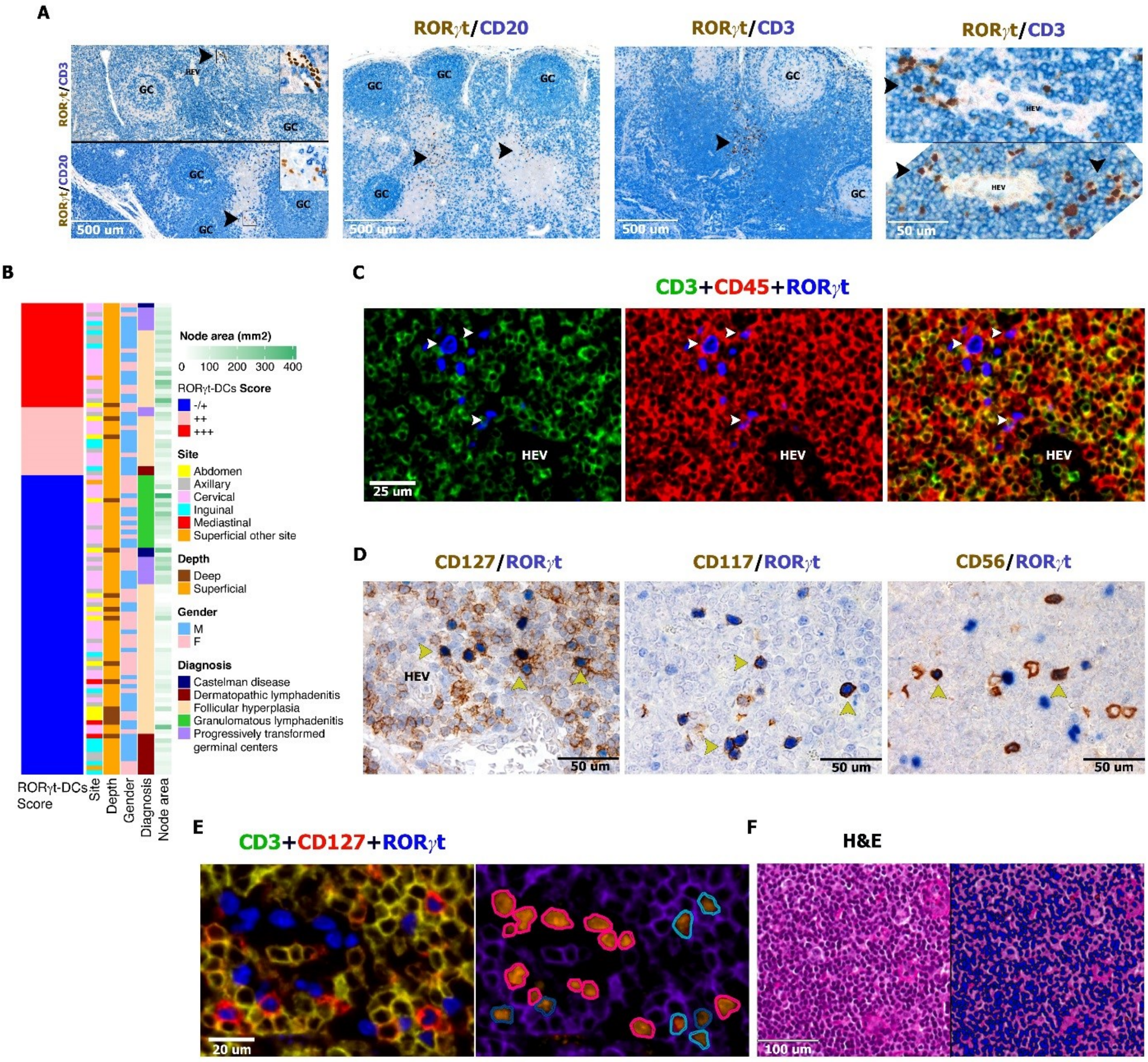


## Supplementary Figure 2

Localization and frequency of RORγt$^{+}$CD3$^{-}$CD127$^{-}$ cells. Sections are from human reactive lymph nodes (n=4) (**A-D**) and stained as labeled. RORγt$^{+}$CD3$^{-}$ large cells are localized in the interfollicular areas (GC=germinal centers) as shown in (**A**, as detailed in insets) through CD20 and CD3 coupled with RORγt, these cells are particularly abundant nearby high endothelial venules (HEV, black arrows). Patients' and samples' characteristics of lymph nodes analyzed for RORγt$^{+}$CD3$^{-}$ cells (with DC-like features) content through semi-quantitative score (n= 104). A fraction of RORγt$^{+}$CD3$^{-}$ cells co-express CD45$^{+}$ (**C**, white arrows). RORγt also identify CD127$^{+}$CD117$^{+}$ and occasionally CD56$^{+}$ ILC3s (**D**, yellow arrows). Quantification of RORγt$^{+}$ cells by a digital image analysis algorithm (QuPath) on IF triple stain is reported in (**E**) (RORγt$^{+}$CD3$^{+}$CD127$^{+}$ T-cells= light blue border, RORγt$^{+}$CD3$^{-}$CD127$^{+}$ILC3= blue border, RORγt$^{+}$CD3$^{-}$CD127$^{-}$large cells= pink border) as a fraction of total nucleated cells as defined by H&E (**F**).

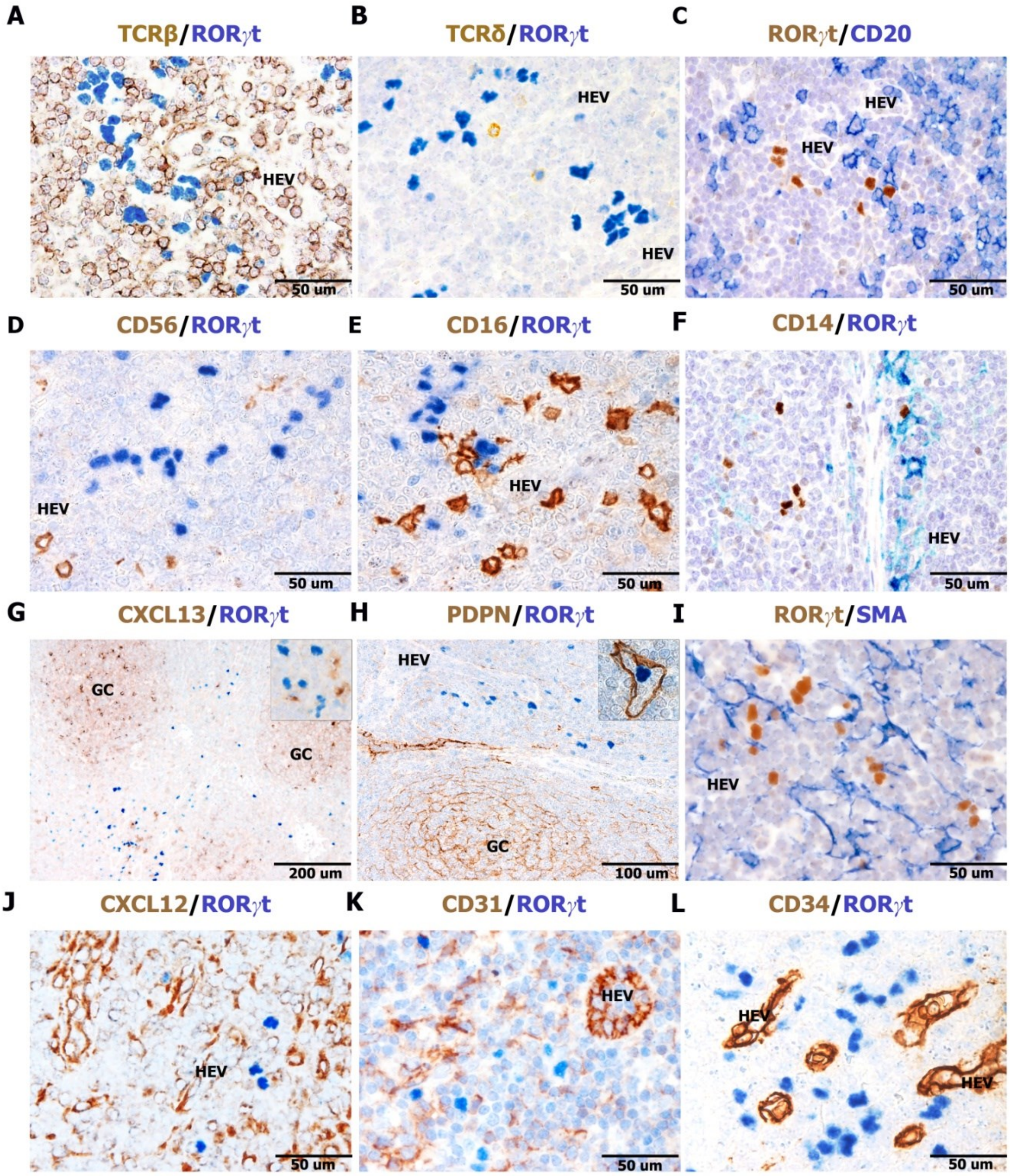


**Supplementary Figure 3**

Phenotype of RORγt$^{+}$CD3$^{-}$ cells in lymph nodes (lineage markers). Sections are from human lymph nodes (**A-L**, n=3) and double stained as indicated by labels. RORγt$^{+}$CD3$^{-}$ cells are negative for TCRβ, TCRδ, CD20, CD56, CD16, CD14, CXCL13, PDPN, SMA, CXCL12, CD31 and CD34. HEV= high endothelial venules, GC= germinal centers.

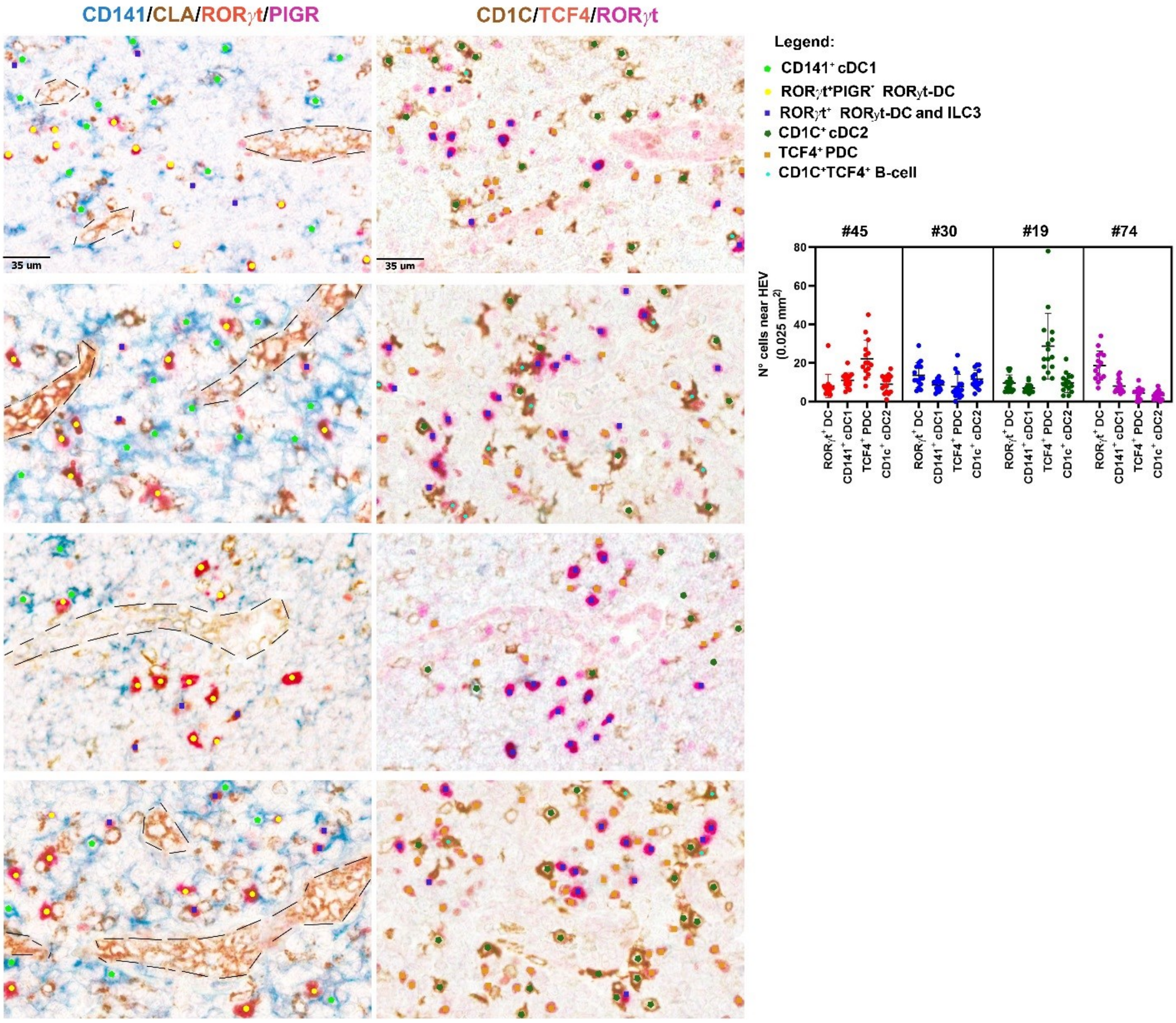


## Supplementary Figure 4

DC microenvironment nearby RORγt-DCs in human lymph nodes. Serial sections are from human lymph nodes (n=4) quadruple (on the left) or triple (on the right) stained as indicated by labels. Geometric figures highlight the populations counted as detailed in the Legend inside the figure. CLA-Heca 452 is used as HEV marker to define the field of count (n=15, 0.025 $mm^2$/each) containing RORγt$^+$PIGR$^+$ RORγt-DCs (combination of fuchsia and red colors, yellow circle). cDC1 DCs are defined by CD141 marker (blue, light green pentagon), whereas cDC2 DCs are defined by CD1C expression (brown, dark green pentagon), with moderate to strong intensity and/or dendritic shape. PDC are TCF4$^+$ (red, orange square). Occasionally B-cells expressing CD1C and TCF4 weakly (combination of brown and red colors, light blue circle) are present. RORγt$^+$ cells on the right panels show the same localization of RORγt-DCs on the left panels. Cells in the left panel not marked as RORγt-DCs (in the absence of PIGR stain) might correspond to ILC3 (blue square).

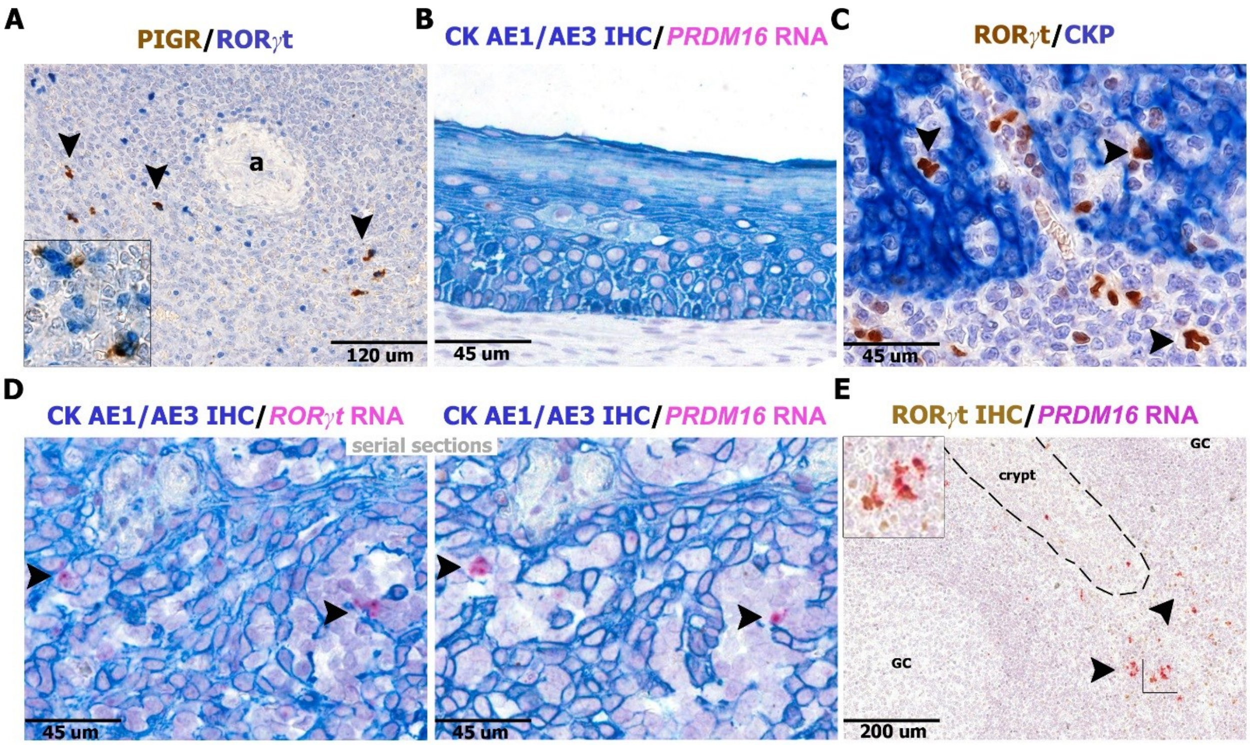


**Supplementary Figure 5**

Localization details of RORγt-DCs in other secondary lymphoid tissues. Sections are from human spleen (**A**) and tonsils (**B-E**, n=3) and double stained as indicated by labels. (**A**) RORγt$^+$PIGR$^+$ -DCs are found near arterioles (a). In tonsil stratified surface epithelium RORγt-DCs are absent as demonstrated by the highly specific transcript *PRDM16* through RNAscope (**B**). Numerous RORγt-DCs are found in reticulated crypt epithelium (**C-D**) confirmed by morphology (**C**) and *RORγt*-*PRDM16* transcripts in serial sections (**D,** same cells are indicated by arrows). RORγt-DCs are also found in interfollicular area of tonsils (**E**). GC= germinal centers.

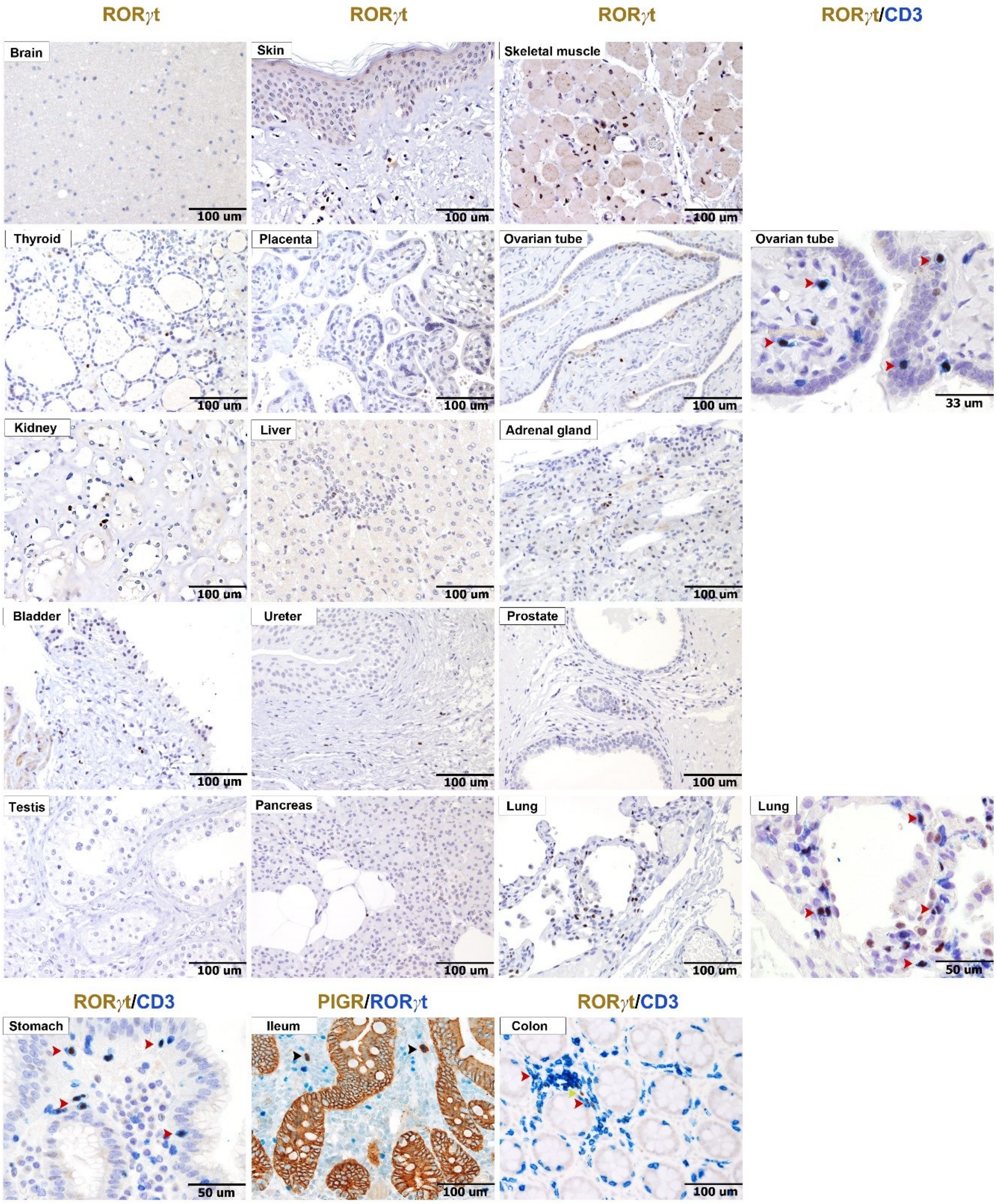


**Supplemental Figure 6**

Absence of RORγt-DCs in most of peripheral tissues. Sections are from human tissues (details in Supplementary Table 4) and immuno-stained as labeled. Nuclear RORγt is largely lacking in all reported tissues; RORγt weakly stains some epithelial cells (ovarian tube and lung) or $CD3^+$ T-cells (as shown by double staining, red arrows) and rare $CD3^-$ ILC3 (yellow arrow in the colon sample). RORγt-DCs are scatter in the lamina propria of ileum (black arrows) as confirmed by PIGR expression. PIGR also stains specifically enterocytes.

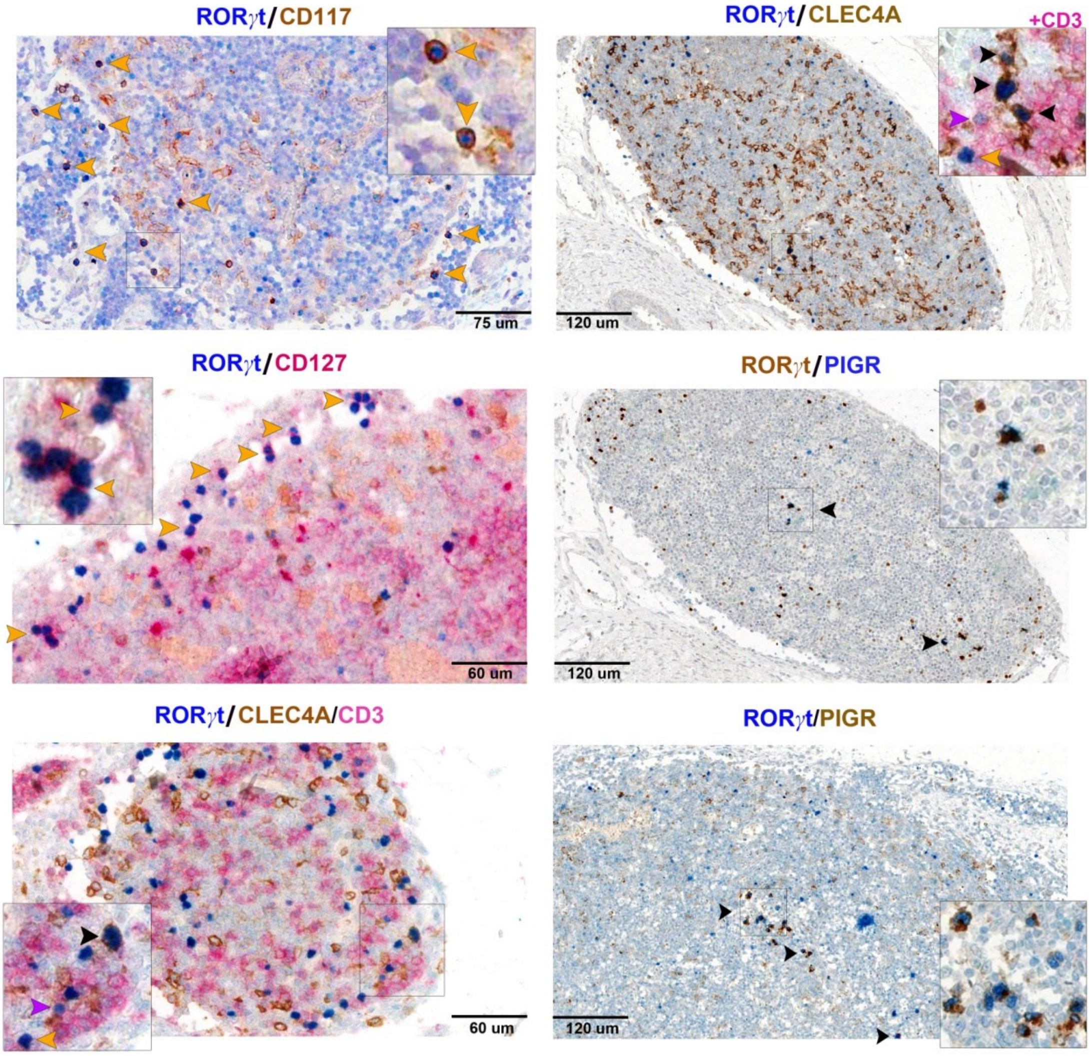


**Supplementary Figure 7**

RORγt$^+$ cell populations in fetal tissues. Sections are from fetal lymph nodes (n=7) and stained as labeled. Numerous RORγt$^+$ cells are found in the human fetal lymph nodes; most of them localize to marginal sinus and correspond to CD127$^+$CD117$^+$CD3$^-$ ILC3 (yellow arrows). More rarely, RORγt-DCs are also identifiable by co-stain for CLEC4A and PIGR (black arrows), occasionally in clusters suggesting proliferative activity. RORγt$^+$CLEC4A$^-$CD3$^+$ T-cells have a weaker RORγt signal compared to ILC3 and RORγt-DCs but are present in all lymph node analyzed (fuchsia arrow in inset).
